# Uncertainty index and stock volatility prediction: evidence from international markets

**DOI:** 10.1186/s40854-022-00361-6

**Published:** 2022-06-08

**Authors:** Xue Gong, Weiguo Zhang, Weijun Xu, Zhe Li

**Affiliations:** 1 School of Business Administration, South China University of Technology, Guangzhou, China; 2 Financial Service Innovation and Risk Management Research Base of Guangzhou, Guangzhou, China; 3 Business School, Nanjing Normal University, Nanjing, China

**Keywords:** Uncertainty index, High-frequency data, Realized variance, Scaled-PCA, C22, G15, G17, G41

## Abstract

This study investigates the predictability of a fixed uncertainty index (UI) for realized variances (volatility) in the international stock markets from a high-frequency perspective. We construct a composite UI based on the scaled principal component analysis (s-PCA) method and demonstrate that it exhibits significant in- and out-of-sample predictabilities for realized variances in global stock markets. This predictive power is more powerful than those of two commonly employed competing methods, namely, PCA and the partial least squares (PLS) methods. The result is robust in several checks. Further, we explain that s-PCA outperforms other dimension-reduction methods since it can effectively increase the impacts of strong predictors and decrease those of weak factors. The implications of this research are significant for investors who allocate assets globally.

## Introduction and literature review

Since the uncertainty and unpredictability of the economic policy and investment environment increase over time, predicting financial market movement is very challenging for scholars and practitioners. Thus, the measurement of the uncertainty in the financial market has attracted enormous attention, e.g., Jurado et al. (2015), Baker et al. (2016) and Huang and Luk (2020). Economic agents typically define uncertainty as the conditional volatility of a disturbance, which is generally unpredictable (Jurado et al. 2015).

In recent years, an increasing number of studies have focused on the linkage between uncertainty and financial market dynamics. For example, several studies have associated the uncertainty therein with stock returns and volatility (Pastor and Veronesi 2012; Li et al. 2020; Megaritis et al. 2021), commodity prices and volatility (Karabulut et al. 2020; Guo et al. 2022), corporate credit spreads (Kaviani et al. 2020), leverage levels (Khan et al. 2020), financial stability (Phan et al. 2021), etc.

Volatility is a well-known indicator for measuring asset price risk. It features a wide range of applications in the fields of finance and economics, such as risk management, asset pricing, and hedging strategies (Chkili 2021; Gong et al. 2022). Moreover, volatility exerts a significant predictive power on potential output growth (Vu 2015). Consequently, an elucidation of the determinants of volatility is quite relevant for investors and policymakers. Volatility is conventionally measured with daily or lower-frequency data [the standard deviation of asset returns, Generalized AutoRegressive Conditional Heteroskedasticity (GARCH)-type model, and so on (Zhang et al. 2021)]. The appearance of the realized volatility (RV), as proposed by Andersen et al. (2001), shortens the distance between the estimated and real volatilities and has been widely adopted in the literature. Compared with the low-frequency one, RV contains richer market information.

Here, we employed five-minute sampling data to construct RV and reduce market microstructure noise to focus on the issue of the high-frequency relationship between the uncertainty index (UI) and realized variance (volatility) in global stock markets. Dissimilar to many studies that had investigated a single extant uncertainty indicator (Liu and Zhang 2015; Megaritis et al. 2021), we explored uncertainty from the equity market, investor, and economic policy levels. Thereafter, we constructed a composite UI based on the scaled principal component analysis (s-PCA) method that was introduced by Huang et al. (2021). Additionally, two well-known competing methods, PCA and the partial least squares (PLS) methods, were employed as competing models.

**Fig. 1:**
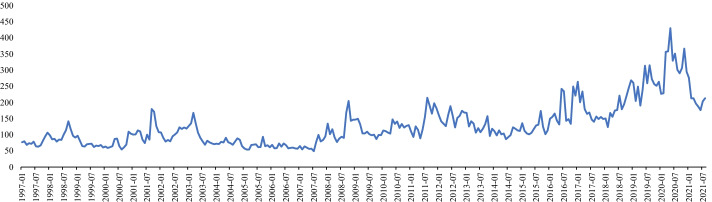
Time dynamics of global economic policy uncertainty index

The motivations were derived from several aspects. Firstly, owing to the increasing trend of international investment, it is necessary to develop a relatively fixed and internationalized risk indicator that monitors market risk dynamics. Particularly, the intensities of the interactions among the global economic entities have grown through the increased liberalization of international trade (Tsai 2017). An increasing number of investors allocate their assets to global markets. Figure 1 shows that the global economic policy uncertainty (EPU) index of Baker et al. (2016) tended to the fluctuant and uncertain international investment environment. Under this condition, monitoring the stock price risk in each market through different indicators might not be an ideal choice because it requires time to separately respond to each market; moreover, it is expensive to simultaneously monitor the stock price risk in each market. Therefore, a relatively fixed indicator that can comprehensively predict the risk of international investment is necessary and convenient for investors to rapidly reach their next investment decisions.

Secondly, only a few studies in the literature focused on the high-frequency relationship between uncertainty and stock volatility. Recent studies offered sufficient evidence confirming that low-frequency uncertainty measures can explain potential financial market volatility. For example, the EPU exerts a significant predictive power on stock volatility (Liu and Zhang 2015; Li et al. 2020), forex volatility (Christou et al. 2018), and European Union allowance futures volatility (Liu et al. 2021). Moreover, Megaritis et al. (2021) argued that the macroeconomic uncertainty sufficiently predicts the U.S. stock volatility. However, the foregoing mainly focused on low-frequency monthly data, even though it is crucial to consider the high-frequency (microcosmic) relationship between uncertainty and volatility. For one thing, many uncertain events, such as the China–US trade war (2018–2019), which was announced by then President Donald Trump on Twitter on August 23, 2019, and the COVID-19 pandemic, which began with the lockdown of Wuhan on January 23, 2020, occur instantaneously. These unexpected events can significantly influence the financial market. A low-frequency investigation cannot readily elucidate this real-time dynamic and random change. For another, compared with the low-frequency volatility, a high-frequency-data-based RV comprises richer trading information and can consistently estimate the true integrated volatility (Andersen et al. 2001). Thus, elucidating the determinants of volatility from the microcosmic perspective is crucial for market participants, particularly short-term investors, to accurately detect financial risks.

Thirdly, many studies in the literature have investigated the predictability of a single UI in a single market (see references in the previous paragraph). It is very interesting to determine whether there is a relatively fixed composite uncertainty indicator that affects international stock markets. This motivation is straightforward and twofold. One, we anticipate a composite index that can reflect a more comprehensive market uncertainty (MU) by capturing uncertainty from different perspectives, such as economic policies and investor behaviors. Compared with a single indicator, the composite index, which is constructed via a dimension-reduction method, could exhibit more robust and outstanding performances in prediction tasks (Neely et al. 2014; Gong et al. 2022). Moreover, a robust composite index is required since this study focuses on international stock market forecasting. For the other fold, we anticipate that a relatively fixed index could influence numerous markets since many studies have documented the strong links, such as volatility co-movement (Cipollini et al. 2015), volatility spillovers (Diebold and Yilmaz 2009), and contagion (Chiang and Wang 2011), among international financial markets. Numerous findings have demonstrated significant volatility spillover effects from the U.S. market on other markets, such as the Pacific-Basin (Ng 2000) and European markets (Baele 2005). Thus, the U.S.-market-based composite UI could potentially impact other markets.

Finally, applying the dimension-reduction technique to the extraction of relevant information from different types of factors has received enormous attention, thus inspiring this study. For example, PCA is generally employed to predict stock volatility (Zhang et al. 2020) and risk premium (Neely et al. 2014). Huang et al. (2015) and Gong et al. (2022) exploited PLS to construct an aligned sentiment index, thereby significantly improving the returns and volatility forecasting, respectively. In a recent study by Huang et al. (2021), an s-PCA method, which demonstrated remarkable predictive performance in macroeconomic forecasting, was developed. Based on this work, Guo et al. (2022) and Yan et al. (2022) confirmed that the s-PCA-based PU index exhibits more powerful predictability on crude oil volatility compared with other competing methods. Moreover, s-PCA is also employed to extract predictive information from macro variables (Huang et al. 2020), technical indicators (He et al. 2021), liquidity indicators (Liao et al. 2021), and investor-attention indicators (Chen et al. 2022). They reported that the s-PCA method improves market returns forecasting. However, it is largely unknown if the s-PCA method is also effective for the prediction of stock volatility, which is fundamentally different from the forecasting of returns (Zhang et al. 2021). Moreover, the application scenarios of the method could be further expanded. Dissimilar to their studies, we applied the s-PCA method to construct a global-level composite uncertainty indicator, which is very beneficial to market participants, as discussed above. Finally and significantly, although Guo et al. (2022) and Yan et al. (2022) argued that the s-PCA method outperforms other competing models, the valid evidence to demonstrate why the s-PCA method is better is still rare, and we will attempt to fill this gap.

Fundamentally, we analyzed the channel from the financial environment uncertainty to the stock price or financial one (Goodell et al. 2020). One theoretical basis derives from increasing the uncertainty about future discount rates, cash flows (dividends), and capital structures. For example, Pastor and Veronesi (2012) revealed that the change in policy or a new policy exerts uncertain impacts on profitability, which will increase the discount rates. Moreover, Megaritis et al. (2021) observed that a significant percentage of stock market fluctuations cannot be explained by fundamentals but only by latent macroeconomic uncertainties. The unexplained component is driven by the uncertainty surrounding future dividend yields. Furthermore, Khan et al. (2020) reported that the listed firms would decrease the level of leverage when the uncertainty increases, thus affecting a firms’ capital structure.

The shocks due to extreme events, such as financial crises and epidemic diseases, account for another channel that explains the predictability of uncertainty on volatility. Naturally, such extreme events occur randomly and intangibly because of the challenge of pre-identifying the factor that generates them. This uncertain factor easily results in irrational trading and contributes to market fluctuations. Academically, numerous studies, e.g., Choudhry (2010) and Wang et al. (2020b), have demonstrated that extreme events can significantly produce violent fluctuations in the stock market. The occurrences of extreme shocks will force market participants to focus more on the financial market dynamics, particularly large asset price fluctuations, and these shocks trigger herding activity and could spread the crisis to neighboring markets (Chiang and Zheng 2010).

To investigate the impacts of uncertainty indices on stock volatilities in 23 relevant international markets, the empirical design was described as follows: the well-known Heterogeneous AutoRegressive-RV (HAR-RV) model (Corsi 2009) was employed as a benchmark model. Next, we employed the PCA, PLS, and s-PCA models to construct the composite uncertainty indices based on a news-based equity market uncertainty (EMU) index (Bakera et al. 2019), investor uncertainty indices measured by market liquidity (Uygur and Taş 2014), implied volatility index (VIX) of the Chicago Board Options Exchange (CBOE) (Deeney et al. 2015), and EPUs from the U.S., U.K., and China (Baker et al. 2016; Huang and Luk 2020). The benchmark model was extended by adding these uncertainty indices, followed by investigating the in-sample and out-of-sample performances. Additionally, several robustness checks were performed, and they supported the result that s-PCA is superior to PCA and PLS. Finally, we discussed why s-PCA outperforms PCA and PLS.

By investigating the predictive power of the proposed composite UI on stock volatilities, this study contributed to the literature in the following aspects. First, a global composite UI based on s-PCA was proposed. This approach is more comprehensive compared with that which was adopted by Yan et al. (2022) and Guo et al. (2022), who developed a composite index employing the s-PCA method on policy-related indices only. The composite index positively affects stock volatility, indicating that a higher uncertainty in the financial environment would increase the price uncertainty, and this is consistent with the theoretical basis and extant studies (Liu and Zhang 2015; Li et al. 2020). Moreover, it exerts significant in- and out-of-sample predictive powers on stock volatility in the 23 markets, although it also exhibited a better and more robust out-of-sample performance than the PCA and PLS methods in most stock markets. Furthermore, this index benefits investors in making decisions, because it is constructed mainly based on the U.S. market data and is relatively fixed.

Second, we observed that VIX is a powerful volatility predictor in most stock markets, and this correlates with the results reported by Wang et al. (2020a); Liang et al. (2020); Megaritis et al. (2021). Additionally, we availed new evidence that the change in VIX (DVIX) exerts a greater short-term predictive power on stock volatility than itself in most markets. Conversely, VIX outperforms DIVX in long-term forecasting. Thus, our results indicated that international investors must focus on different indicator forms (itself or its change) for different investment horizons (short-term or long-term). Moreover, high-frequency EPUs exhibit weak predictability on stock volatility, disagreeing with much extant evidence from monthly frequencies, e.g., Liu and Zhang (2015) and Li et al. (2020). This indicates that it is not rational to apply daily EPUs to the identification of market risk movement, which should be a warning to market participants.

Finally, this study empirically answered the question regarding why s-PCA outperforms PCA and PLS via time-varying loadings. We demonstrated that the main contributors of the PCA, PLS, and s-PCA factors are markedly different. More specifically, the loadings of the PCA factors exhibited generally equal relevance. Thus, its predictability would be reduced in the presence of strong and weak predictors. Further, the PLS method can effectively identify the main predictors but cannot reasonably assign weights. Contrarily, s-PCA is a superior method because it can effectively extract relevant predictive information and extract weak factors by placing a higher (lower) weight on the powerful (weak) predictors, thus ensuring a better prediction performance.

The remainder of this paper is organized as follows: “Measurement” section presents the measurements; “Methodology” section introduces the methodologies; and “Empirical analysis” section reports the empirical results, including the in-sample, out-of-sample, longer forecast horizon, and robustness analyses. “Predictability analyses” section further analyzes the difference in the predictability methods from the microcosmic perspective. Finally, our conclusions are reported in “Conclusion” section.

## Measurement

This section introduces the measurement methods, including RV and UIs, employed in this study. We demonstrated the uncertainty measures from three aspects, including MU, investor uncertainty, and EPU.

### Realized variance

The utilization of high-frequency data to model volatility is a well-known and widely accepted approach because it could be a good proxy for real volatility. Realized variance,^1^ indicated as *RV*, the sum of the squared log-returns, as defined by Andersen et al. (2001), is a simple, efficient, and consistent estimator of volatility. To overcome the influence of microstructure noise, sampling every five minutes is a common method. Following this, *RV* on the trading day, *t*, is given by the following:

1 $$\begin{aligned} R V_{t}=\sum _{j=1}^{M_{t}} r_{t, j}^{2}, \end{aligned}$$

where $r_{t, j}=\log \left( p_{t, j}\right) -\log \left( p_{t, j-1}\right)$ is the logarithmic returns from time, $j-1$ to *j*; $p_{t,j}$ refers to the closing price on the *j*th five-minute point in the trading periods; and $M_t$ denotes the number of five-minute intervals in the *t*th trading period.

### Uncertainty variable

Two aspects are generally considered when selecting the uncertainty measures. One involves focusing on the high-frequency relationship, and the other involves exploring a relatively fixed UI that exerts a significant predictive power on *international* stock markets. Thus, the following uncertainty measures were employed. They are mainly derived from the American market since it is the biggest and most developed capital market worldwide.

#### Equity market uncertainty

Facing the big data area, the media account for the main source of information for the public. Different types of participants, including retail and institutional investors, managers, and policymakers, exist in this field. Thus, we cannot ignore the information from the media that are related to MU. Accordingly, we employed the newspaper-based equity market uncertainty index (**EMU**), which was proposed by Bakera et al. (2019), to capture the uncertainty reported by the media. EMU was constructed employing the scaled frequency counts of newspaper articles that contain the following three types of sets: economic, economy, and financial; stock market, equity, equities, etc.; and volatility, volatile, risk, etc.

#### Investor uncertainty

We postulated that investor psychology, which dominates investors’ behaviors, can be viewed as a source of uncertainty in the financial market for two reasons. One, investor psychology is unpredictable because it changes with the information that are available to the investor. Thus, investor psychology can reflect uncertain information from the market via investors. Secondly, investor sentiment and attention are good measures for capturing investors’ cognitive biases (Baker and Wurgler 2006; Da et al. 2011). Investor sentiment is regarded as the propensity to generally speculate (display optimism or pessimism) markets. Put differently, investor sentiment comprises future expectations. Investor attention is defined as a scarce cognitive resource. Extreme events are expected to increase investors’ attention via Internet activities, e.g., the search volume on Google. Thus, investor psychology must be the source of uncertainty in the financial market.

Considering the availability of high-frequency data, the first employed investor uncertainty was the CBOE volatility index (**VIX**) because it is a proxy of investor sentiment (Deeney et al. 2015), which is also employed as an uncertainty measure (Wang et al. 2020a; Megaritis et al. 2021). Considering that VIX is a popular and powerful factor that affects the financial market, we further focused on the changes therein, indicated by **DVIX**, to capture the change in investor uncertainty. Another measure is the change in the trading volume (**VOL**) of the National Association of Securities Dealers Automated Quotations (NASDAQ) composite index. This measure is regarded as an information flow (Zhang et al. 2021), and is a good proxy of market liquidity, which adequately reflects investor sentiment (Baker and Wurgler 2006; Uygur and Taş 2014).

#### Economic policy uncertainty

Aldy and Viscusi (2014) reported that environmental risks might comprise the most relevant policy-related applications of the economics of risk and uncertainty. The linkage between EPU and economic activities has been widely proven, e.g., Liu and Zhang (2015); Li et al. (2020). However, the studies focused on low-frequency analysis; the microcosmic evidence is lacking. We selected EPUs from the U.S. (**USEPU**), U.K. (**UKEPU**), and China (**CNEPU**) since they constitute powerful and influential countries globally. Another reason is the availability of high-frequency data. The newspaper-based USEPU and UKEPU indexes were proposed by Baker et al. (2016) who measure uncertainty by calculating the number of keywords in leading newspapers, such as economic or economy; uncertain or uncertainty. Although Baker et al. (2016) also introduced CNEPU, we employed the measure proposed by Huang and Luk (2020) because it is based on more comprehensive materials, including ten influential newspapers in mainland China.

## Methodology

### Dimension reduction methods

A single UI could be limited to predicting the stock volatility in international markets; thus, a composite index is required because it can capture uncertainty from a more comprehensive perspective. Moreover, considering all the UIs in a “kitchen sink” model, it is easy to achieve in-sample over-fitting and poor out-of-sample performances (Huang et al. 2015, 2021). To address it, this study introduced three types of dimension-reduction methods to construct composite indexes.

Assuming that there were *N* uncertainty indicators, $u_{i,t}$ for $i=1, \cdots , N$, that are relevant but imperfect predictor variables of the target variable (RV) denoted by $U_t=\left( u_{1, t}, u_{2, t},\cdots , u_{N, t}\right) ^{\prime }$ for $t=1, \cdots , T$, where *T* refers to the number of observations. $U= \{\mathrm {EMU, DVIX, VOL, USEPU, UKEPU, CNEPU}\}$ for the following analyses, as well as the definition of each $u_{i,t}$, are presented in Table 1. Notably, we employed DVIX here, rather than VIX, to consider the stationarity of time series, which aims to avoid incorrect statistical inferences. Following the convention, we standardized each predictor in set *U* before constructing these composite uncertainty indicators.

#### PCA and s-PCA techniques

The oldest and most commonly employed approach for combining predictors into a lower-dimensional linear space is the (**PCA**) model, which could preserve the covariance structure among these factors (Gu et al. 2020). Mathematically, the PCA model extracts diffusion indexes as linear combinations of the predictors, i.e., set *U* in this study, via the following equation:

2 $$\begin{aligned} u_{i,t}=\mu _i+\lambda _{i}^{\prime } F_{t}^{\mathrm {P C A}}+\epsilon _{i, t}, \quad i=1, 2, \cdots , N, \quad t=1, 2, \ldots , T, \end{aligned}$$

where $F_{t}^{\mathrm {P C A}}$ is the PCA diffusion indexes that were extracted from $U_t=\left( u_{1, t}, u_{2, t};\cdots , u_{N, t}\right) ^{\prime }$, which is a *K*-dimensional vector ($K<<N$), $\lambda$, is the *K*-dimensional parameter to be estimated; and $e_{i, t}$ is the idiosyncratic noise term.

Although PCA is a well-known dimension-reduction technique that has been widely employed in the literature, it is limited by its negligence of the ultimate statistical objective. An improved target-driven dimension-reduction method is the s-PCA method that was recently proposed by Huang et al. (2021); it scales each predictor variable with its predictive slope on the to-be-predicted target. This method is implemented by the following two steps: first, we generated a panel of scaled predictors, $\left( {\hat{\theta }}_{1} u_{1, t},{\hat{\theta }}_{1} u_{2, t}, \ldots , {\hat{\theta }}_{N} u_{N, t}\right)$, in which the coefficient, ${\hat{\theta }}_{i}$, was the estimated slope from regressing the target variable on the *i*th uncertainty predictor, $u_{i, t}$, as follows:

3 $$\begin{aligned} \log (RV_{t})=\theta _{i, 0}+\theta _{i} u_{i, t}+\epsilon _{i, t}, \quad i=1, 2, \cdots , N. \end{aligned}$$

Second, similar to Eq. (2), we applied PCA to $\left( {\hat{\theta }}_{1} u_{1, t},{\hat{\theta }}_{1} u_{2, t}, \ldots , {\hat{\theta }}_{N} u_{N, t}\right)$ to extract the factors and forecast the target variable. Compared with PCA, Huang et al. (2021) argued that the s-PCA exhibited several advantages: (i) s-PCA could distinguish between the target-relevant and -irrelevant latent factors when the factors are strong, while PCA could not; (ii) s-PCA could extract the signals from a large amount of noise, while PCA failed to do that, thus resulting in biased forecasts even when all the factors were weak.

Subsequently, we investigated two cases involving the use of s-PCA: in the first case, we employed the first principal component to measure a composite UI, denoted by **s-FPCA**. In the other case, we employed a weight s-PCA, following Gong et al. (2022), and defined as follows:

4 $$\begin{aligned} \mathrm {UI}^\mathrm {s-PCA}=\sum _{i=1}^{M}\left( \mathrm {PC}_{i}^{\mathrm {s-PCA}} \cdot {eigen}_{i}\right) / \sum _{j=1}^{M} {eigen}_{j}, \end{aligned}$$

where $\mathrm {PC_{i}}^\mathrm {s-PCA}$ is the *i*th principal component, ${eigen }_{i}$ is its eigenvalue, and *M* is the total number of principal components. Compared with s-FPCA, the weighted s-PCA index (**s-PCA**) comprises more predictive information that could be useful since it is screened by the target variable.

#### PLS technique

Another supervised learning technique is the PLS (**PLS**) method, which can separate the irrelevant component from the proxy variables and extract the predictive information for the forecasting task (Huang et al. 2015). Following Huang et al. (2015) and Gong et al. (2022), PLS can be implemented via the following two steps:

In the first step, we ran the time-series regressions *N* times, where *N* is the number of basic uncertainty proxies. More specifically, each uncertainty predictor variable, $u_{i, t}$, regressed on a constant and logarithmic *RV*. Namely,

5 $$\begin{aligned} u_{i, t}=\phi _{i, 0}+\phi _{i} \log (RV_{t})+\epsilon _{i, t}, \quad t=1, 2, \ldots , T, \end{aligned}$$

where the loading $\phi _{i}$ captures the sensitivity of each $u_i$ to the uncertainty measure that was instrumented by *RV*.

In the second step, *T* cross-sectional regressions were run. For each time period, *t*, we regressed $u_i$ on the estimated coefficient, ${\hat{\phi }}_{i}$, in the regression 5 and obtained the following:

6 $$\begin{aligned} u_{i, t}=\psi _{t}+\mathrm {UI}_{t}^{\mathrm {PLS}} \hat{\phi }_{i}+\varepsilon _{i, t}, \quad i=1, 2, \cdots , N, \end{aligned}$$

where the slope of this regression, $\mathrm {UI}_{t}^{\mathrm {PLS}}$, is the estimated PLS uncertainty index.

Notably, we employed contemporaneous regression in the target-related equations, Eq. (3) and (5), differing from the application in the return predictions of Huang et al. (2015) and Huang et al. (2021). This is because the volatility was highly autocorrelated, dissimilar to the asset returns. The predictive information regarding the volatility must exert a potential predictive power on one-step-ahead volatilities. Moreover, the volatility model below considers the historical information on the volatility. Thus, focusing on the contemporaneous target variable can prevent the overlap of information between the volatility and uncertainty indicators.

This study investigated whether there was a *fixed* uncertainty indicator that significantly impacted stock volatility in international markets. Thus, the target variables in Eqs. (3) and (5) were set as the logarithmic RVs of the Dow Jones Industrial Average (DJIA) stock index. This is because the U.S. market is the biggest and most developed capital market. Moreover, the well-known volatility spillover effects examined the shocks from the U.S. to other markets, such as the European equity (Baele 2005) and Pacific-Basin (Ng 2000) markets. Therefore, we assumed that the composite uncertainty indicator, which is driven by the volatility of the U.S. stock market, might effectively predict other equity markets.

### Predictive regression model and its extension

To investigate whether UI is an effective factor for predicting stock volatility, we first set the **HAR-RV** model that was proposed by Corsi (2009) as the *benchmark model*. This model is based on the heterogeneous market hypothesis, where the heterogeneity derives from the differences in time horizons, i.e., the different types of market participants, such as high- and low-frequency traders, exert different impacts on future volatility. The HAR-RV model is formulated as follows:

7 $$\begin{aligned} R V_{t+h}=\alpha _0+\alpha ^{(d)} R V_{t}+\alpha ^{(w)} R V_{t}^{(5)}+\alpha ^{(m)} R V_{t}^{(22)}+\epsilon _{t+h}, \end{aligned}$$

where $R V_{t}^{(m)}=\sum _{n=1}^{m} R V_{t-n+1} / m$, and *h* denote the forecast horizon.

Afterward, following Liang et al. (2020); Zhang et al. (2021) among others, we incorporated UI into the HAR-RV model. Apparently, the **HAR-RV-UI** model was specified as follows:

8 $$\begin{aligned} R V_{t+h}=\alpha _0+\alpha ^{(d)} R V_{t}+\alpha ^{(w)} R V_{t}^{(5)}+\alpha ^{(m)} R V_{t}^{(22)}+\beta \mathrm {UI}_{t}+\epsilon _{t+h}, \end{aligned}$$

where the key variable UI $\in${EMU, VIX, DVIX, VOL, USEPU, UKEPU, CNEPU, PCA, PLS, s-FPCA, s-PCA}. In the following, we focused on the coefficient, $\beta$, since its significance reflects the predictability of UI.

Regarding the estimations of the parameters of the predictive regression models (7) and (8), we employed the *logarithmic* RV to ensure that the distributions were more approximately Gaussian, following the report of Paye (2012), Gong et al. (2022) and others. This prevented achieving a misleading statistical inference in the ordinary least squares (OLS) estimation. Notably, we employed the information available only up to time *t* to predict the target variable in time $t+h$, to avoid the look-ahead bias in the out-of-sample analysis. More specifically, when employing the composite UI to predict RV, we calculated PCA, PLS, s-FPCA, and s-PCA recurrently with only the in-sample data to avoid the usage of the out-of-sample information for the prediction of the out-of-sample RV.

### Forecast combination

Although this study mainly focused on the relationship between UI and stock volatility, we also compared the predictive performances of the dimension-reduction methods and forecast-combination methods since the latter is widely employed as the competing models, e.g., Guo et al. (2022) and Yan et al. (2022). The forecast combinations employed all the predictive information from each predictor (Set *U*) and combined them to obtain the final prediction. This method can be mathematically described as follows Timmermann (2006) and Weiss et al. (2018): First, we ran the HAR–RV–UI model (8) on each uncertainty indicator $u_i$ ($\in U$) to obtain the *individual forecasts*

9 $$\begin{aligned} {\widehat{RV}}_{n,t+1}={\hat{\alpha }}_{0,n}+{\hat{\alpha }}_n^{(d)} {\widehat{RV}}_{n,t}+{\hat{\alpha }}_n^{(w)} {\widehat{RV}}_{n,t}^{(5)})+{\hat{\alpha }}_n^{(m)} {\widehat{RV}}_{n,t}^{(22)})+{\hat{\beta }}_n \mathrm {UI}_{n,t}, \end{aligned}$$

where, ${\hat{\alpha }}_{0,n}$, ${\hat{\alpha }}_n^{(d)}$, ${\hat{\alpha }}_n^{(w)}$, ${\hat{\alpha }}_n^{(m)}$, and ${\hat{\beta }}_n$ are the estimated coefficients from model (8) of the *n*th uncertainty indicator employing the information up to time $t-1$, and *n*=1, 2, $\cdots$, *N*. Thereafter, the final prediction was obtained by combining the individual forecasts based on some weight schemes, as follows:

10 $$\begin{aligned} {\widehat{RV}}_{t\mid t-1}^C=\sum _{n=1}^{N} \omega _{n, t-1} {\widehat{RV}}_{n, t\mid t-1}, \end{aligned}$$

where *C* is the combination style determined by the weight, $\omega _{t-1}$, given at time, $t-1$.

Three types of classical forecast combinations were employed as the competing models. The first simple method is the mean combination (**MC**) obtained by averaging all the individual forecasts as follows:

11 $$\begin{aligned} {\widehat{RV}}_{t \mid t-1}^{MC}=\frac{1}{N} ({\widehat{RV}}_{t \mid t-1, 1}+ {\widehat{RV}}_{t \mid t-1, 2}+\cdots + {\widehat{RV}}_{t \mid t-1, N}), \end{aligned}$$

i.e., $$\omega _{n, t-1}=1 / N$$.

The second simple-weighted method is the median combination (**MEDC**) obtained from the median values of the individual forecasts, as exhibited below:

12 $$\begin{aligned} {\widehat{RV}}_{t \mid t-1}^{MEDC}=Median \{{\widehat{RV}}_{t \mid t-1, 1}, {\widehat{RV}}_{t \mid t-1, 2}, \cdots , {\widehat{RV}}_{t \mid t-1, N} \}. \end{aligned}$$

The winsorized mean (**WMC**) is the final combination, which handles outliers employing a softer line. This method caps outliers at a certain level, and it is specified as follows:

13 $$\begin{aligned} {\widehat{RV}}_{t \mid t-1}^{WMC}=\frac{1}{N}\left[ \lambda N {\widetilde{RV}}_{t \mid t-1,\lambda N+1}+\sum _{n=\lambda N+1}^{N-\lambda N}{\widetilde{RV}}_{t \mid t-1,n} +\lambda N {\widetilde{RV}}_{t \mid t-1,N-\lambda N}\right] , \end{aligned}$$

where $\lambda$ is also a trim factor, i.e., the top/bottom 100$\cdot$ $\lambda$% are winsorized, that takes the value of 0.1 in the empirical analysis; ${\widetilde{RV}}_{i}$ is the *i*th statistic by increasing order in $\{{\widehat{RV}}_n\}_{n=1}^{N}$. This measure involves taking the ($\lambda N$)th smallest and ($\lambda N$)th largest forecasts and equating them to the $\left( \lambda N+1\right)$th smallest and $\left( \lambda N+1\right)$th largest forecasts, respectively.

### Out-of-sample regression mechanism and evaluation criteria

Out-of-sample predictability could change with time since many extreme events, such as the sub-prime crisis in 2008 and the COVID-19 pandemic in 2020, occurred during our sampling periods. Following Catania and Proietti (2020), we addressed this employing a rolling window regression method, which is a common technique for evaluating stability and prediction accuracies in time-series forecasting. More specifically, we split the full sample, *T*, into initial train data (in-sample) with a fixed window length, *W*, and test data (out-of-sample) with $T-W$ observations. This fixed window method replaces one old observation and a new one. In the empirical analysis, we employed a four-year window, i.e., $W=1000$, to conduct the investigations. As alternative robustness checks, $W=2000$ and 3000 were discussed.

To assess the out-of-sample relative performance of the UI model concerning the benchmark model, following Huang et al. (2015) and Neely et al. (2014), the out-of-sample $R^2$ ($R_{\mathrm {OS}}^{2}$) was employed to evaluate the out-of-sample performance. It is given by the following:

14 $$\begin{aligned} R_{\mathrm {OS}}^{2}=1-\frac{\sum _{t=1}^{T_{\mathrm {OS}}}I_{t}^{\mathrm {C}}\left( RV_{r,t}-RV_{f,t}^{\mathrm {U}}\right) ^{2}}{\sum _{t=1}^{T_{\mathrm {OS}}}I_{t}^{\mathrm {C}}\left( RV_{r,t}-RV_{f,t}^{\mathrm {B}}\right) ^{2}}, \quad \mathrm {C}=\mathrm {Full}, \text{ Expansions }, \text{ Contractions }, \end{aligned}$$

where $RV_{r,t}$ refers to the actual RV, $RV_{f,t}^{\mathrm {B}}$ and $RV_{f,t}^{\mathrm {U}}$ are the fitted values from the benchmark (7) and UI (8) models, respectively, $T_{\mathrm {OS}}$ denotes the out-of-sample size, and $I_{t}^{\mathrm {c}}$ is an indicator function whose value is 1 if day *t* belongs to the periods of *C* and 0 otherwise. Computing $R_{\mathrm {OS}}^{2}$ separately during economic expansions and contractions clarifies whether UI exerts a significant out-of-sample predictive power over the different economic periods.

We expected $R_{\mathrm {OS}}^{2}$ to be significantly positive from a statistical perspective, i.e., the mean square prediction error (MSPE) from the competing model is expected to be less than that of the benchmark model, indicating that UI can improve the out-of-sample predictive performance. We exploited an approximately normal test that was developed by Clark and West (2007) for equal predictive accuracy. The null (alternative) hypothesis states that the benchmark model has equal or less (larger) MSPE with the competing model, corresponding to $H_0$: $R_{\mathrm {OS}}^{2} \le 0$ against $H_A$: $R_{\mathrm {OS}}^{2} > 0$. To realize it, we regressed the time series ${\hat{f}}_{t}$, formulated by

15 $$\begin{aligned} {\hat{f}}_{t}=\left( RV_{r,t}-RV_{f, t}^{\mathrm {B}}\right) ^{2}-\left[ \left( RV_{r,t}-RV_{f, t}^{\mathrm {U}}\right) ^{2}-\left( RV_{f, t}^{\mathrm {B}}-RV_{f, t}^{\mathrm {U}}\right) ^{2}\right] , \end{aligned}$$

on a constant and calculated the *t* statistic corresponding to the constant coefficient. Thereafter, the *t* statistic from a one-tailed (right) test was employed for the statistical decision.

## Empirical analysis

This section discusses the predictability of UIs on RVs of international stock markets based on in- and out-of-sample analyses. Moreover, we investigated its predictive power on longer horizons. Finally, several robustness checks were designed to analyze the performances of the uncertainty indicators under different conditions.

### Data and statistical analyses

The information regarding the single uncertainty variables, including the abbreviations, definitions, periods, and data sources of the variables, are presented in Table 1. Moreover, we focused on 23 stock markets globally, e.g., the U.S., Australia, Belgium, Brazil, Canada, China, Denmark, Euro Area, Finland, France, Germany, Hong Kong, India, Italy, Japan, Mexico, Norway, Pakistan, South Korea, Spain, Sweden, Switzerland, and the U.K., covering five continents, as well as developed and developing markets. Notably, these markets were the main focus of the literature. We obtained the high-frequency RV data of stock indexes from the realized library.^2^

**Table 1:** Definition of uncertainty variables

Variable	Definition	Full period	Trading days	Data source
EMU	Equity market uncertainty index for the United States (Log)	2001/1/1–2021/08/31	7548	Public website 1
VIX	The CBOE volatility index (Log)	2001/1/2–2021/08/31	5199	Public website 2
DVIX	The CBOE volatility index (Log change)	2001/1/2–2021/08/31	5199	Public website 2
VOL	Volume of NASDAQ stock index (investor sentiment proxy, Log change)	2001/1/2–2021/08/31	5199	Public website 2
USEPU	Economic policy uncertainty for the United States (Log change)	2001/1/1–2021/08/31	7548	Public website 1
UKEPU	Economic policy uncertainty for the United Kingdom (Log change)	2001/1/1–2021/08/31	7548	Public website 1
CNEPU	Economic policy uncertainty for Chinese mainland (Log change)	2001/1/1–2021/08/31	7548	Public website 3

This table reports the information of uncertainty indexes. The public websites are accessed by following websites: Public website 1 (Economic policy uncertainty for the US and the UK): https://www.policyuncertainty.com/, Public website 2 (Yahoo Finance): https://finance.yahoo.com/, Public website 3 (Economic policy uncertainty for China): https://economicpolicyuncertaintyinchina.weebly.com/

Table 2 presents description statistics of the RVs. Most stock indexes covered the period between January 1, 2001, and August 31, 2021. Some exhibited a shorter interval owing to data availability. The autocorrelation coefficients ($\rho$) revealed that RVs were highly dependent, thus indicating the rationality of modeling the HAR-RV model. Moreover, the Jarque and Bera (1987) statistic (JB-stat) rejects the null hypothesis, indicating that all the time series did not follow the normal distribution. Thus, it was necessary to take the logarithm transformation in the empirical analysis to avoid misleading statistical inferences. The augmented Dickey–Fuller (ADF) statistic, which was developed by Cheung and Lai (1995), indicated that all the time series were stationary, and this is a sufficient condition for conducting econometric analyses. Finally, the difference in the observations (Obs.) indicated that each market had a different number of trading days.

**Table 2:** Description statistics of realized variances

ID	Country/Region	Symbol	Name	Full period	Mean	St.D.	Max	Min	$\rho _{1}$	$\rho _{5}$	JB-stat	ADF	Obs.
					($\times$ 0.01)						($\times$ 10$^5$)		
1	Australia	AORD	All Ordinaries	2001/01/02–2021/08/31	0.607	0.403	0.691	0.010	0.709	0.633	3.515***	− 8.096***	5218
2	Belgium	BFX	Bell 20 Index	2001/01/02–2021/08/31	0.821	0.477	0.607	0.020	0.810	0.689	0.671***	− 7.702***	5269
3	India	BSESN	S&P BSE Sensex	2001/01/01–2021/08/31	0.980	0.630	1.221	0.020	0.741	0.564	5.442***	− 8.577***	5119
4	Brazil	BVSP	BVSP BOVESPA Index	2001/01/02–2021/08/31	1.090	0.589	0.769	0.023	0.772	0.636	1.059***	− 7.901***	5082
5	America	DJI	Dow Jones Industrial Average	2001/01/02–2021/08/31	0.845	0.625	0.929	0.014	0.790	0.678	1.312***	− 7.361***	5177
6	France	FCHI	CAC 40	2001/01/02–2021/08/31	0.981	0.598	0.716	0.017	0.822	0.712	0.577***	− 7.749***	5273
7	Italy	FTMIB	FTSE MIB	2009/06/01–2021/08/31	0.950	0.494	0.522	0.009	0.752	0.611	0.179***	− 7.033***	3105
8	UK	FTSE	FTSE 100	2001/01/02–2021/08/31	0.914	0.614	1.030	0.012	0.734	0.631	1.953***	− 7.457***	5210
9	Germany	GDAXI	DAX	2001/01/02–2021/08/31	1.051	0.677	0.767	0.020	0.830	0.726	0.384***	− 7.098***	5234
10	Canada	GSPTSE	S&P/TSX Composite index	2002/05/02–2021/08/31	0.692	0.604	1.714	0.012	0.728	0.630	36.566***	− 6.713***	4833
11	HongKong	HSI	HANG SENG Index	2001/01/02–2021/08/31	0.842	0.440	0.661	0.021	0.738	0.638	1.398***	− 6.172***	5059
12	Spain	IBEX	IBEX 35 Index	2001/01/02–2021/08/31	1.046	0.570	0.742	0.020	0.791	0.650	0.450***	− 7.69***	5239
13	South Korea	KS11	Korea Composite Stock Price Index	2001/01/02–2021/08/31	0.874	0.519	0.771	0.012	0.826	0.718	0.888***	− 7.581***	5090
14	Pakistan	KSE	Karachi SE 100 Index	2001/01/01–2021/08/31	0.843	0.513	0.608	0.001	0.691	0.533	0.236***	− 8.748***	5028
15	Mexico	MXX	IPC Mexico	2001/01/02–2021/08/31	0.759	0.448	0.723	0.019	0.603	0.494	1.891***	− 7.698***	5183
16	Japan	N225	Nikkei 225	2001/01/04–2021/08/31	0.867	0.492	0.620	0.014	0.756	0.597	0.659***	− 8.295***	5038
17	Denmark	OMXC20	OMX Copenhagen 20 Index	2005/10/03–2021/08/31	0.932	0.615	1.103	0.026	0.649	0.539	3.554***	− 6.548***	3951
18	Finland	OMXHPI	OMX Helsinki All Share Index	2005/10/03–2021/08/31	0.851	0.633	1.499	0.020	0.686	0.578	11.069***	− 6.655***	3991
19	Sweden	OMXSPI	OMX Stockholm All Share Index	2005/10/03–2021/08/31	0.804	0.581	1.026	0.015	0.720	0.621	2.335***	− 6.777***	3990
20	Norway	OSEAX	Oslo Exchange All-share Index	2001/09/03–2021/08/31	0.958	0.640	1.403	0.026	0.698	0.607	5.706***	− 7.485***	4980
21	China	SSEC	Shanghai Composite Index	2001/01/02–2021/08/31	1.074	0.668	0.654	0.019	0.785	0.639	0.198***	− 6.123***	4994
22	Switzerland	SSMI	Swiss Stock Market Index	2001/01/03–2021/08/31	0.787	0.510	0.745	0.025	0.829	0.720	1.720***	− 7.853***	5175
23	Euro Area	STOXX50E	EURO STOXX 50	2001/01/02–2021/08/31	1.056	0.682	1.041	0.001	0.781	0.664	0.952***	− 8.063***	5266

This table reports summary descriptions of realized variance in 23 international stock markets. St.D., Max, Min, and Obs donate standard deviation, maximum value, minimum value, and the number of observations, respectively. $\rho _{i}$ refers to auto-correlation coefficient with *i* lags. The JB-stat and ADF represent the Jarque–Bera statistic (Jarque and Bera 1987) and Augmented Dickey–Fuller unit root test statistic (Cheung and Lai 1995), respectively. The null hypothesis of the Jarque–Bera test is that sample data has skewness and kurtosis following a normal distribution. The null hypothesis of the Augmented Dickey–Fuller unit root test is that there is a unit root in the time series

**Fig. 2:**
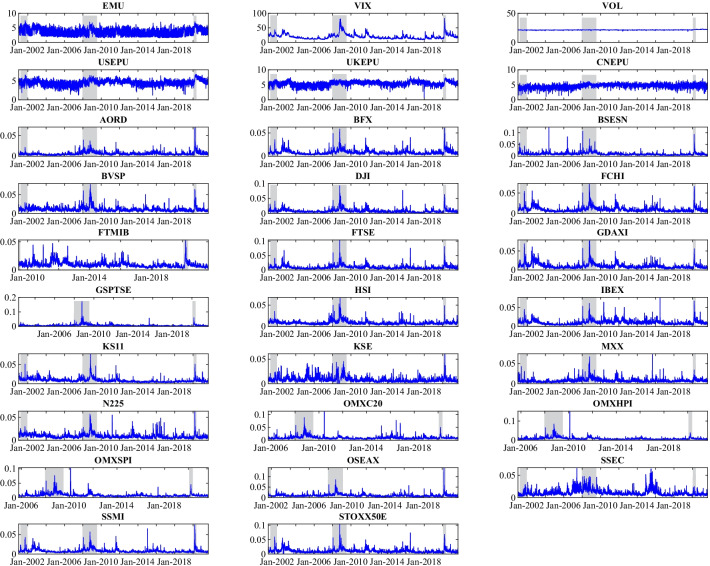
Time series of realized variances and uncertainty indices

Figure 2 shows the time dynamics of the uncertainty indicators and RVs. The shaded area highlights the National Bureau of Economic Research (NBER)-dated economic recession periods.^3^ Evidently, RVs increased during the economic contractions, particularly during the 2008 sub-prime crisis and the COVID-19 pandemic. This result is consistent with the trends of EMU and VIX. However, it was challenging to determine whether there was a potential relationship between EPUs and the economic cycle since EPUs fluctuate frequently and irregularly. Moreover, regarding the VOL, we observed a relatively subdued tendency. Finally, we noted that several stock indexes, which the economic cycle could not capture, fluctuated acutely. For example, the Chinese stock market (SSEC) fluctuated greatly and frequently before the 2008 sub-prime crisis and was shocked between 2015 and 2016 owing to the well-known 2015–2016 Chinese stock market turbulence. Additionally, the Pakistani stock market (KSE) exhibited continuous fluctuations over time. These findings indicate that these stock markets were not steady and could cause many challenges to the prediction task.

### In-sample analysis

Table 3 reports the in-sample results of the one-step-ahead forecasts ($h=1$). For the single UIs, we observed that EMU, VIX, DVIX, and VOL significantly impacted RVs in most stock markets. More specifically, EMU and VIX performed poorly only in the Chinese market (SSEC). VOL could not predict stock volatility in the American (DJI) and Pakistan (KSE) markets. Surprisingly and interestingly, the change in VIX (DVIX) performed well in all the stock markets. What’s more, DVIX delivered a better predictive performance than VIX according to the magnitude of the adjusted $R^2$, indicating that the changes in VIX exerted more power to capture the market dynamics than itself. Moreover, the positive coefficients indicated that volatility increases with uncertainty. This result is consistent with some findings regarding the relationship between uncertainty and volatility, e.g., Li et al. (2020) and Megaritis et al. (2021). The results indicate that the uncertainty information about the U.S. market could effectively impact the stock volatility in many international stock markets.

However, the predictive abilities of the EPU indexes were weak. Each EPU exerts significant impacts on several markets ($\le$4) from the perspective of the number of significant results. From the significant-level perspective, most of the results were not statistically significant or were significant at a low level (10% or 5%). These findings indicated that EPUs were not strong predictor variables for predicting stock volatility. This contradicts the arguments of Li et al. (2020) and Liu and Zhang (2015), who observed a significant relationship between EPU and stock volatility. This might be because we utilized high-frequency data, while they utilized a monthly frequency.

The composite UIs demonstrated a robust and significant predictive power on all stock markets except for PLS of the Chinese market (SSEC). This result was expected since the composite indices were derived from many single uncertainty indicators exhibiting significant predictabilities on RV in international stock markets. Moreover, the highest adjusted $R^2$s often appear in the s-PCA index, indicating that this composite uncertainty indicator exerted the best in-sample predictability. Notably, the composite UIs exhibited very close predictability with DVIX, which is the best volatility factor in the single uncertainty indicators. Thus, the predictive ability of the composite UIs might mainly derive from DVIX.

**Table 3:** In-sample results for one-step-ahead forecasts ($h=1$)

Asset	EMU	VIX	DVIX	VOL	USEPU	UKEPU	CNEPU	PCA	PLS	s-FPCA	s-PCA
AORD	0.018***	0.007***	0.964***	0.284***	0.010	0.014*	− 0.013*	0.061***	0.174***	0.099***	0.207***
	(4.242)	(10.147)	(10.764)	(10.260)	(1.232)	− 1.810	(− 1.876)	(12.259)	(9.071)	(11.109)	(12.206)
	[59.58%]	[60.34%]	[61.43%]	[60.62%]	[59.43%]	[59.45%]	[59.44%]	[61.18%]	[60.21%]	[61.51%]	**[61.85%]**
BFX	0.011***	0.009***	0.847***	0.087***	− 0.001	0.010*	0.006	0.034***	0.108***	0.086***	0.169***
	(3.180)	(10.921)	(16.274)	(4.224)	(− 0.100)	(1.780)	(1.086)	(9.105)	(7.142)	(16.457)	(16.511)
	[73.30%]	[74.20%]	[74.99%]	[73.46%]	[73.25%]	[73.26%]	[73.25%]	[73.98%]	[73.70%]	[75.08%]	**[75.12%]**
BSESN	0.014***	0.003***	0.505***	0.082***	− 0.003	0.002	− 0.007	0.023***	0.088***	0.052***	0.105***
	(3.738)	(5.270)	(8.424)	(3.779)	(− 0.431)	(0.274)	(− 1.159)	(5.824)	(5.410)	(8.700)	(8.986)
	[68.94%]	[69.33%]	[69.63%]	[69.17%]	[68.84%]	[68.84%]	[68.85%]	[69.42%]	[69.38%]	[69.75%]	**[69.80%]**
BVSP	0.013***	0.004***	0.459***	0.068***	0.004	0.004	0.008	0.024***	0.085***	0.047***	0.095***
	(3.785)	(7.125)	(8.151)	(3.153)	(0.521)	(0.569)	(1.306)	(5.956)	(5.749)	(8.423)	(8.670)
	[60.34%]	[60.69%]	[60.85%]	[60.34%]	[60.23%]	[60.23%]	[60.25%]	[60.58%]	[60.50%]	[60.87%]	**[60.91%]**
DJI	0.023***	0.017***	1.027***	− 0.013	0.010	− 0.011	0.010	0.030***	0.146***	0.103***	0.198***
	(4.969)	(13.673)	(14.238)	(− 0.554)	(1.278)	(− 1.457)	(1.376)	(6.609)	(7.458)	(14.190)	(13.964)
	[68.72%]	[70.06%]	**[70.14%]**	[68.56%]	[68.57%]	[68.57%]	[68.56%]	[68.84%]	[68.93%]	[70.13%]	[70.08%]
FCHI	0.012***	0.009***	0.967***	0.128***	− 0.007	0.008	0.004	0.039***	0.121***	0.098***	0.195***
	(3.046)	(11.099)	(17.834)	(5.502)	(− 1.152)	(1.169)	(0.655)	(10.108)	(7.589)	(18.021)	(18.280)
	[73.24%]	[74.18%]	[75.20%]	[73.56%]	[73.19%]	[73.19%]	[73.18%]	[73.99%]	[73.66%]	[75.23%]	**[75.32%]**
FTMIB	0.010***	0.006***	0.864***	0.081***	− 0.015	0.000	0.000	0.036***	0.105***	0.086***	0.168***
	(2.139)	(5.745)	(13.969)	(2.503)	(− 1.627)	(0.007)	(0.044)	(6.723)	(5.080)	(14.375)	(14.208)
	[63.45%]	[64.11%]	[65.83%]	[63.73%]	[63.41%]	[63.39%]	[63.39%]	[64.50%]	[64.19%]	[66.03%]	**[66.04%]**
FTSE	0.014***	0.015***	0.929***	0.195***	0.012	0.000	− 0.008	0.048***	0.151***	0.095***	0.193***
	(3.367)	(12.912)	(12.008)	(7.150)	(1.510)	(− 0.005)	(− 1.056)	(9.886)	(8.015)	(12.243)	(12.938)
	[66.21%]	[67.68%]	[67.57%]	[66.41%]	[66.15%]	[66.14%]	[66.14%]	[66.83%]	[66.38%]	[67.60%]	**[67.77%]**
GDAXI	0.010***	0.008***	0.901***	0.165***	− 0.007	0.008	− 0.002	0.041***	0.119***	0.092***	0.185***
	(2.406)	(9.511)	(16.054)	(6.612)	(− 1.006)	(1.212)	(− 0.279)	(10.097)	(7.082)	(16.412)	(16.894)
	[73.66%]	[74.34%]	[75.19%]	[74.05%]	[73.63%]	[73.63%]	[73.62%]	[74.37%]	[74.01%]	[75.25%]	**[75.36%]**
GSPTSE	0.008*	0.008***	0.758***	0.057**	0.021**	0.009	0.004	0.033***	0.085***	0.077***	0.152***
	(1.744)	(9.199)	(10.299)	(2.011)	(2.464)	(1.068)	(0.566)	(6.462)	(4.255)	(10.487)	(10.449)
	[66.91%]	[67.67%]	[67.92%]	[67.03%]	[66.93%]	[66.90%]	[66.89%]	[67.39%]	[67.16%]	[67.97%]	**[67.99%]**
HSI	0.016***	0.006***	0.555***	0.083***	0.013**	0.003	− 0.003	0.029***	0.107***	0.056***	0.112***
	(4.522)	(7.935)	(9.473)	(3.575)	(2.213)	(0.531)	(− 0.468)	(7.406)	(6.858)	(9.509)	(9.746)
	[63.52%]	[64.20%]	[64.31%]	[63.49%]	[63.38%]	[63.35%]	[63.36%]	[64.09%]	[63.97%]	[64.51%]	**[64.57%]**
IBEX	0.009***	0.005***	0.762***	0.102***	− 0.010	0.011*	− 0.002	0.033***	0.096***	0.078***	0.154***
	(2.717)	(7.283)	(14.134)	(4.484)	(− 1.644)	(1.818)	(− 0.453)	(8.778)	(6.653)	(14.397)	(14.737)
	[73.77%]	[74.14%]	[75.02%]	[73.91%]	[73.74%]	[73.75%]	[73.73%]	[74.27%]	[74.01%]	[75.08%]	**[75.13%]**
KS11	0.013***	0.004***	0.729***	0.070***	0.000	− 0.008	0.003	0.026***	0.105***	0.073***	0.143***
	(3.618)	(6.600)	(13.748)	(3.388)	(− 0.016)	(− 1.407)	(0.449)	(7.148)	(6.731)	(13.771)	(13.854)
	[76.29%]	[76.51%]	[77.34%]	[76.28%]	[76.21%]	[76.22%]	[76.22%]	[76.59%]	[76.55%]	[77.43%]	**[77.45%]**
KSE	0.011***	0.002***	0.200***	0.000	0.009	0.009	0.006	0.011***	0.048***	0.020***	0.038***
	(2.376)	(3.091)	(2.730)	(− 0.002)	(0.968)	(1.109)	(0.799)	(2.164)	(2.356)	(2.689)	(2.675)
	[57.77%]	**[57.91%]**	[57.86%]	[57.79%]	[57.73%]	[57.73%]	[57.73%]	[57.79%]	[57.80%]	[57.81%]	[57.81%]
MXX	0.011***	0.004***	0.463***	0.075***	0.009	− 0.001	0.004	0.022***	0.074***	0.047***	0.096***
	(2.643)	(6.567)	(6.297)	(3.094)	(1.152)	(− 0.144)	(0.555)	(4.711)	(4.151)	(6.432)	(6.664)
	[51.91%]	[52.58%]	[52.67%]	[52.25%]	[51.85%]	[51.84%]	[51.84%]	[52.38%]	[52.03%]	[52.66%]	**[52.71%]**
N225	0.019***	0.006***	0.966***	0.146***	0.001	0.000	− 0.006	0.042***	0.150***	0.097***	0.194***
	(4.890)	(8.725)	(13.716)	(5.997)	(0.088)	(0.043)	(− 0.916)	(8.991)	(8.562)	(13.756)	(14.098)
	[66.69%]	[67.05%]	[68.46%]	[66.65%]	[66.52%]	[66.52%]	[66.53%]	[67.24%]	[66.98%]	[68.52%]	**[68.64%]**
OMXC20	0.021***	0.009***	0.699***	0.213***	0.000	0.011	− 0.002	0.051***	0.170***	0.072***	0.150***
	(4.339)	(10.406)	(8.926)	(7.281)	(− 0.021)	(1.250)	(− 0.258)	(9.003)	(7.975)	(9.254)	(9.940)
	[59.82%]	[60.95%]	[60.89%]	[60.26%]	[59.60%]	[59.61%]	[59.60%]	[60.96%]	[60.46%]	[61.12%]	**[61.33%]**
OMXHPI	0.018***	0.010***	0.826***	0.215***	− 0.015	0.012	0.013*	0.051***	0.162***	0.085***	0.173***
	(3.789)	(10.536)	(13.415)	(6.857)	(− 1.573)	(1.306)	(1.710)	(10.555)	(8.164)	(13.886)	(14.795)
	[69.84%]	[70.79%]	[71.20%]	[70.29%]	[69.75%]	[69.74%]	[69.74%]	[70.72%]	[70.30%]	[71.31%]	**[71.48%]**
OMXSPI	0.015***	0.010***	0.977***	0.194***	− 0.018**	0.013	0.013*	0.053***	0.160***	0.100***	0.200***
	(3.295)	(10.343)	(15.875)	(6.640)	(− 1.994)	(1.448)	(1.694)	(10.577)	(8.156)	(16.226)	(16.811)
	[71.09%]	[72.07%]	[73.09%]	[71.52%]	[71.04%]	[71.03%]	[71.03%]	[72.11%]	[71.60%]	[73.17%]	**[73.31%]**
OSEAX	0.013***	0.011***	0.840***	0.169***	0.012	0.004	0.001	0.044***	0.132***	0.085***	0.172***
	(2.990)	(12.967)	(10.092)	(6.547)	(1.610)	(0.492)	(0.177)	(8.823)	(6.848)	(10.272)	(10.801)
	[61.81%]	[63.02%]	[63.08%]	[61.95%]	[61.75%]	[61.73%]	[61.73%]	[62.44%]	[62.00%]	[63.15%]	**[63.28%]**
SSEC	− 0.004	0.001	0.486***	0.053**	0.011	0.013*	0.010	0.019***	0.021	0.048***	0.094***
	(− 1.087)	(1.336)	(8.216)	(2.244)	(1.447)	(1.893)	(1.385)	(4.853)	(1.369)	(8.176)	(8.171)
	[71.66%]	[71.71%]	[72.15%]	[71.74%]	[71.67%]	[71.67%]	[71.66%]	[71.93%]	[71.79%]	[72.23%]	**[72.23%]**
SSMI	0.013***	0.008***	0.788***	0.112***	− 0.007	0.002	− 0.002	0.033***	0.116***	0.080***	0.158***
	(4.018)	(11.926)	(15.591)	(6.114)	(− 1.307)	(0.438)	(− 0.414)	(10.033)	(8.168)	(15.685)	(15.989)
	[77.09%]	[77.83%]	[78.52%]	[77.25%]	[77.01%]	[77.01%]	[77.00%]	[77.64%]	[77.44%]	[78.57%]	**[78.64%]**
STOXX50E	0.014***	0.011***	1.004***	0.203***	− 0.001	0.003	0.000	0.048***	0.150***	0.102***	0.207***
	(3.049)	(10.652)	(14.644)	(7.074)	(− 0.111)	(0.353)	(0.002)	(10.296)	(7.874)	(14.848)	(15.613)
	[63.75%]	[64.49%]	[65.20%]	[63.92%]	[63.68%]	[63.68%]	[63.67%]	[64.29%]	[63.88%]	[65.24%]	**[65.40%]**

This table reports slope coefficient $\beta$ in model (8) for one-step-ahead forecasts in international stock markets. The definitions of uncertainty indicators such as EMU and VIX are shown in Table 1. The asset list is reported in Table 2. Newey and West (1987) adjusted *t* statistics are reported in parenthesis. The adjusted $R^2$s are shown in the square

### Out-of-sample analysis

Table 4 presents the out-of-sample results. The bold font highlights the significantly positive $R_{\mathrm {OS}}^{2}$s, and the underline font highlights the highest one.^4^ We observed that EMU, VIX, DVIX, and VOL exhibited *insignificant* the out-of-sample predictive abilities in only a few stock markets. More specifically, EMU exhibited poor ability in forecasting RVs in Italy (FTMIB), Canada (GSPTSE), Pakistan (KSE), and China (SSEC). VIX and DVIX did not perform well in only SSEC and KSE, respectively. Additionally, VOL could not effectively predict the stock volatilities in Brazil (BVSP), America (DJI), Pakistan (KSE), and China (SSEC). The terrible performances in China and Pakistan were predictable because the volatilities of both markets fluctuated greatly and frequently (see Figure 2). Moreover, compared with VIX, we noted that DVIX exerted stronger predictive ability in most markets based on its greater $R_{\mathrm {OS}}^{2}$s. Thus, DVIX is a better indicator for identifying the potential movement of stock volatility compared with VIX. This finding meaningfully supplements the extant literature investigating the short-term impact of VIX on stock volatility, e.g., Wang et al. (2020a) and Liang et al. (2020). However, most EPUs performed poorly and even had negative $R_{\mathrm {OS}}^{2}$ values in most cases, indicating that the high-frequency relation between EPU and stock volatility was *not* significant.

The composite UIs exhibited significant predictability on RVs in all the markets except for s-FPCA of KSE. Thus, compared with the single uncertainty indicators, the composite indices delivered more robust prediction results. What’s more, the s-PCA methods performed better than PCA and PLS according to the magnitude of $R_{\mathrm {OS}}^{2}$, indicating that the s-PCA method exerted a higher power to capture prediction information from single uncertainty indicators and incorporate lesser noise. Although the composite indexes exhibited the highest $R_{\mathrm {OS}}^{2}$ (the underlined ones) occasionally, their prediction accuracy was inferior to those of DVIX in some cases, implying that the predictability was mainly derived from DVIX.

**Table 4:** Out-of-sample results for one-step-ahead forecast

Asset	EMU	VIX	DVIX	VOL	USEPU	UKEPU	CNEPU	PCA	PLS	s-FPCA	s-PCA
AORD	**0.53%**	**2.77%**	**6.00%**	**2.89%**	**0.11%**	− 0.06%	− 0.07%	**3.16%**	**4.45%**	**7.13%**	**7.45%**
	(4.533***)	(8.291***)	(8.881***)	(9.856***)	(2.316***)	(0.646)	(0.515)	(8.906***)	(9.934***)	(9.751***)	(9.063***)
BFX	**0.15%**	**4.26%**	**7.00%**	**0.37%**	− 0.09%	0.03%	− 0.07%	**2.52%**	**2.01%**	**5.84%**	**5.87%**
	(2.691***)	(8.805***)	(11.455***)	(3.989***)	(− 0.092***)	(1.388)	(− 0.584)	(8.525***)	(7.898***)	(10.009***)	(8.864***)
BSESN	**0.45%**	**2.29%**	**2.13%**	**0.29%**	− 0.04%	− 0.14%	− 0.13%	**1.15%**	**1.48%**	**2.24%**	**2.38%**
	(3.936***)	(5.110***)	(7.643***)	(3.463***)	(0.933)	(− 0.406)	(0.145)	(6.706***)	(6.476***)	(7.621***)	(7.148***)
BVSP	**0.19%**	**1.19%**	**1.56%**	0.05%	− 0.09%	− 0.11%	− 0.02%	**1.09%**	**0.92%**	**1.64%**	**1.83%**
	(3.028***)	(6.119***)	(7.101***)	(1.603)	(− 0.563)	(− 0.373)	(1.224)	(6.090***)	(5.507***)	(7.058***)	(7.124***)
DJI	**0.42%**	**5.19%**	**4.86%**	− 0.10%	− 0.06%	− 0.02%	− 0.09%	**1.47%**	**1.27%**	**3.65%**	**3.72%**
	(3.760***)	(13.450***)	(11.224***)	(0.476)	(0.252)	(0.738)	(− 0.241)	(7.087***)	(6.595***)	(9.197***)	(9.699***)
FCHI	**0.23%**	**4.28%**	**7.39%**	**0.78%**	− 0.08%	− 0.09%	− 0.09%	**2.46%**	**2.23%**	**6.19%**	**6.27%**
	(3.269***)	(9.837***)	(12.820***)	(5.38***)	(0.324)	(− 0.405)	(− 0.421)	(9.044***)	(8.606***)	(11.417***)	(10.202***)
FTMIB	− 0.22%	**2.66%**	**6.28%**	**0.37%**	− 0.04%	− 0.05%	− 0.19%	**1.96%**	**1.33%**	**5.95%**	**5.61%**
	(1.082)	(6.025***)	(9.154***)	(2.838***)	(0.477)	(0.250)	(− 1.785*)	(5.555***)	(4.788***)	(8.576***)	(7.594***)
FTSE	**0.13%**	**5.41%**	**5.22%**	**1.24%**	− 0.04%	− 0.08%	− 0.11%	**2.74%**	**2.67%**	**5.90%**	**6.18%**
	(2.323***)	(10.676***)	(8.920***)	(7.033***)	(1.016)	(− 1.354)	(− 0.814)	(7.980***)	(8.361***)	(9.209***)	(7.727***)
GDAXI	**0.03%**	**3.00%**	**6.18%**	**1.01%**	− 0.19%	− 0.08%	− 0.15%	**2.39%**	**1.79%**	**5.87%**	**5.76%**
	(2.044***)	(9.507***)	(11.867***)	(5.986***)	(− 0.019)	(− 0.030)	(− 0.913)	(8.595***)	(7.683***)	(11.616***)	(10.359***)
GSPTSE	− 0.05%	**2.74%**	**3.03%**	**0.04%**	0.03%	− 0.13%	− 0.14%	**1.27%**	**0.85%**	**2.86%**	**2.94%**
	(1.333)	(8.438***)	(8.360***)	(1.719*)	(1.388)	(− 1.490)	(− 0.138)	(5.822***)	(5.158***)	(7.867***)	(7.569***)
HSI	**0.40%**	**2.01%**	**2.87%**	**0.28%**	− 0.09%	− 0.06%	− 0.18%	**1.35%**	**1.65%**	**2.51%**	**2.80%**
	(3.755***)	(6.119***)	(8.201***)	(3.233***)	(− 0.908)	(0.214)	(0.119)	(5.701***)	(6.419***)	(7.285***)	(6.850***)
IBEX	**0.28%**	**2.02%**	**5.42%**	**0.40%**	− 0.06%	− 0.03%	− 0.07%	**2.33%**	**1.68%**	**4.40%**	**4.83%**
	(3.437***)	(8.057***)	(11.262***)	(4.015***)	(0.921)	(0.527)	(0.342)	(8.686***)	(7.535***)	(10.286***)	(9.835***)
KS11	**0.56%**	**3.34%**	**5.08%**	**0.17%**	− 0.17%	− 0.15%	− 0.19%	**1.86%**	**2.19%**	**3.93%**	**4.60%**
	(4.530***)	(7.378***)	(11.561***)	(2.714***)	(− 1.568)	(− 0.945)	(− 1.496)	(8.060***)	(8.129***)	(9.656***)	(9.931***)
KSE	− 0.02%	**0.32%**	− 0.03%	− 0.19%	0.00%	− 0.25%	− 0.12%	**0.01%**	**0.05%**	0.00%	**0.06%**
	(1.265)	(3.041***)	(1.458)	(− 0.602)	(1.431)	(− 0.405)	(− 0.458)	(1.921*)	(1.995**)	(1.564)	(2.047**)
MXX	**0.29%**	**0.94%**	**1.09%**	− 0.04%	− 0.17%	− 0.09%	− 0.09%	**0.60%**	**0.68%**	**1.09%**	**1.26%**
	(3.754***)	(7.481***)	(5.513***)	(1.583)	(− 0.790)	(− 0.142)	(− 0.117)	(4.546***)	(4.962***)	(5.308***)	(5.496***)
N225	**0.53%**	**1.72%**	**6.96%**	**0.84%**	− 0.15%	− 0.03%	− 0.13%	**2.97%**	**3.23%**	**7.30%**	**7.68%**
	(4.364***)	(6.896***)	(10.880***)	(5.789***)	(− 1.475)	(0.574)	(− 0.224)	(8.366***)	(8.892***)	(10.583***)	(9.211***)
OMXC20	**0.59%**	**2.21%**	**3.26%**	**0.82%**	− 0.13%	− 0.03%	− 0.11%	**2.66%**	**2.58%**	**3.59%**	**3.96%**
	(3.977***)	(7.072***)	(6.406***)	(5.509***)	(− 0.264)	(0.745)	(− 0.880)	(6.939***)	(7.189***)	(6.422***)	(5.847***)
OMXHPI	**0.69%**	**5.44%**	**5.91%**	**0.74%**	− 0.21%	− 0.04%	− 0.05%	**4.46%**	**3.07%**	**6.17%**	**6.39%**
	(4.669***)	(8.937***)	(9.984***)	(4.516***)	(− 0.563)	(0.580)	(0.870)	(10.199***)	(8.616***)	(9.701***)	(8.445***)
OMXSPI	**0.49%**	**5.00%**	**7.82%**	**0.44%**	− 0.23%	0.01%	− 0.09%	**4.70%**	**2.83%**	**7.86%**	**7.99%**
	(3.946***)	(8.493***)	(10.955***)	(3.865***)	(− 1.006)	(1.359)	(− 0.258)	(9.227***)	(8.038***)	(10.314***)	(8.68***)
OSEAX	**0.12%**	**4.76%**	**4.69%**	**0.92%**	− 0.17%	− 0.07%	− 0.10%	**3.20%**	**2.47%**	**4.76%**	**4.94%**
	(2.784***)	(9.969***)	(8.638***)	(6.424***)	(− 0.474)	(− 0.316)	(− 0.452)	(8.387***)	(7.911***)	(8.497***)	(7.583***)
SSEC	− 0.10%	− 0.19%	**2.18%**	− 0.05%	− 0.11%	0.03%	− 0.15%	**0.57%**	**0.30%**	**1.70%**	**1.58%**
	(− 0.375)	(2.047***)	(8.525***)	(0.783)	(− 0.034)	(1.231)	(0.541)	(4.624***)	(3.089***)	(7.585***)	(7.163***)
SSMI	**0.25%**	**2.67%**	**6.93%**	**0.87%**	− 0.02%	− 0.10%	0.01%	**2.82%**	**2.25%**	**5.60%**	**5.93%**
	(3.218***)	(8.670***)	(11.697***)	(5.551***)	(0.399)	(− 0.967)	(1.007)	(9.206***)	(8.155***)	(10.216***)	(9.618***)
STOXX50E	**0.14%**	**4.31%**	**5.36%**	**1.30%**	− 0.17%	− 0.03%	− 0.14%	**2.65%**	**2.34%**	**5.51%**	**5.53%**
	(2.572**)	(10.364***)	(11.029***)	(6.727***)	(0.289)	(0.209)	(− 1.170)	(9.295***)	(8.860***)	(11.057***)	(9.853***)

This table reports out-of-sample results for one-step-ahead forecast based on models (7) and (8). The bold font highlights the significantly positive $R_{\mathrm {OS}}^{2}$s according to the Clark and West (2007) test, and the underline font highlights the biggest one. The statistics of Clark and West (2007) test are shown in parentheses, which corresponds to test $H_0$: $R_{OS}^2\le 0$ against $H_A$: $R_{OS}^2$>0. *, ** and *** refer to statistical significance at 10%, 5% and 1% level, respectively

### Comparison with the forecast combination models

We compared the prediction accuracy of the dimension-reduction methods and the forecast combination methods based on the model confidence set (MCS) test of Hansen et al. (2011). The results based on the Tmax statistic, which were evaluated by MSPE and the mean absolute error (MAE), are presented in Table 5.^5^ We set the confidence level to be 90%, indicating that a model was excluded from MCS if the *p*-value was <0.1. The *p*-values were obtained based on 10,000 block bootstraps. The results demonstrated that the maximum *p*-value generally appeared in the s-(F)PCA model, indicating that the s-(F)PCA model exhibited better prediction accuracies in different evaluation indicators and different stock markets (except for KSE) than the competing models from the statistical perspective.

**Table 5:** MCS test based on T-MAX statistics for competing models

Asset	MSPE								MAE							
	HAR-RV	PCA	PLS	sFPCA	sPCA	MC	MEDC	WMC	HAR-RV	PCA	PLS	sFPCA	sPCA	MC	**MEDC**	**WMC**
AORD	0.000	0.000	0.000	**1.000**	0.286	0.000	0.000	0.000	0.000	0.290	0.263	**1.000**	**1.000**	**1.000**	0.000	0.814
BFX	0.000	0.000	0.000	**1.000**	0.873	0.000	0.000	0.000	0.000	0.000	0.000	**1.000**	0.781	0.000	0.000	0.000
BSESN	0.000	0.125	0.623	**1.000**	**1.000**	0.603	0.000	0.000	0.000	0.287	0.310	**1.000**	**1.000**	0.980	0.000	0.204
BVSP	0.000	0.461	0.294	**1.000**	**1.000**	0.381	0.000	0.000	0.000	0.489	0.300	**1.000**	**1.000**	**1.000**	0.000	0.997
DJI	0.000	0.000	0.000	**1.000**	**1.000**	**1.000**	0.000	0.135	0.000	0.000	0.000	**1.000**	**1.000**	**1.000**	0.000	0.238
FCHI	0.000	0.000	0.000	**1.000**	0.804	0.000	0.000	0.000	0.000	0.000	0.000	**1.000**	0.384	0.000	0.000	0.000
FTMIB	0.000	0.000	0.000	0.000	**1.000**	0.000	0.000	0.000	0.000	0.000	0.000	0.176	**1.000**	0.000	0.000	0.000
FTSE	0.000	0.000	0.000	**1.000**	0.289	0.000	0.000	0.000	0.000	0.000	0.000	**1.000**	0.621	0.000	0.000	0.000
GDAXI	0.000	0.000	0.000	0.702	**1.000**	0.000	0.000	0.000	0.000	0.000	0.000	**1.000**	0.820	0.000	0.000	0.000
GSPTSE	0.000	0.000	0.000	**1.000**	**1.000**	0.100	0.000	0.000	0.000	0.000	0.000	**1.000**	**1.000**	0.186	0.000	0.000
HSI	0.000	0.154	0.439	**1.000**	**1.000**	**1.000**	0.000	0.529	0.000	0.104	0.642	**1.000**	**1.000**	**1.000**	0.000	**1.000**
IBEX	0.000	0.000	0.000	**1.000**	0.000	0.000	0.000	0.000	0.000	0.000	0.000	**1.000**	0.217	0.000	0.000	0.000
KS11	0.000	0.000	0.000	**1.000**	0.000	0.000	0.000	0.000	0.000	0.000	0.000	**1.000**	0.000	0.000	0.000	0.000
KSE	0.930	0.995	**1.000**	**1.000**	0.967	**1.000**	**1.000**	**1.000**	**1.000**	0.965	0.860	0.851	0.965	**1.000**	**1.000**	**1.000**
MXX	0.000	0.000	0.227	**1.000**	**1.000**	**1.000**	0.000	0.896	0.000	0.126	0.000	**1.000**	**1.000**	**1.000**	0.000	0.968
N225	0.000	0.000	0.000	**1.000**	0.000	0.000	0.000	0.000	0.000	0.000	0.000	**1.000**	0.882	0.000	0.000	0.000
OMXC20	0.000	0.736	0.714	**1.000**	**1.000**	**1.000**	0.000	0.393	0.000	0.000	0.000	**1.000**	**1.000**	0.183	0.000	0.000
OMXHPI	0.000	0.000	0.000	**1.000**	0.128	0.000	0.000	0.000	0.000	0.000	0.000	**1.000**	0.174	0.000	0.000	0.000
OMXSPI	0.000	0.000	0.000	**1.000**	0.438	0.000	0.000	0.000	0.000	0.000	0.000	0.839	**1.000**	0.000	0.000	0.000
OSEAX	0.000	0.000	0.000	**1.000**	**1.000**	0.170	0.000	0.000	0.000	0.109	0.000	**1.000**	**1.000**	**1.000**	0.000	0.330
SSEC	0.000	0.000	0.000	0.266	**1.000**	0.000	0.000	0.000	0.000	0.000	0.000	0.490	**1.000**	0.000	0.000	0.000
SSMI	0.000	0.000	0.000	**1.000**	0.198	0.000	0.000	0.000	0.000	0.000	0.000	**1.000**	0.135	0.000	0.000	0.000
STI	0.000	0.000	0.000	**1.000**	**1.000**	0.103	0.000	0.000	0.000	0.146	0.437	**1.000**	**1.000**	**1.000**	0.000	0.287
STOXX50E	0.000	0.000	0.000	**1.000**	0.939	0.000	0.000	0.000	0.000	0.000	0.000	**1.000**	0.907	0.000	0.000	0.000

This table reports MCS test (*p*-value) result based on TMAX statistics proposed by Hansen et al. (2011). MSPE and MAE are mean square prediction error and mean absolute error respectively. The null hypothesis of MCS test is that arbitrary two models in the model set have equal predictive ability. *p*-value>0.1 denotes that the corresponding models are included in MCS for the confidence of 90%. The numbers with overstriking (*p*-value=1) highlight the best predictive models

### Longer forecast horizon analyses

To determine whether the predictability of UIs was persistent, we further investigated the out-of-sample performance on longer forecasts horizons. More specifically, we set horizon *h* as 3, 6, and 12, and Table 6 presents the corresponding results. To conserve space, we only reported the results of $R_{\mathrm {OS}}^{2}$, where the bold font indicates that the value was significantly positive, following the test by Clark and West (2007) and the underline font denotes that the value was the highest in the corresponding row. Overall, most UIs exerted a significant predictive power on longer horizons, although their impacts decreased with the increasing forecast horizon (except for several particular cases). This result indicated the persistence of their predictive abilities. Interestingly, VIX performed better on the longer prediction horizons because many of the highest $R_{\mathrm {OS}}^{2}$s (the underlined ones) appeared. Thus, considering the long view, VIX was more effective for forecasting stock volatility concerning other uncertainty indicators.

**Table 6:** Out-of-sample predictability for longer horizons

Asset	Horizon	EMU (%)	VIX (%)	DVIX (%)	VOL (%)	USEPU (%)	UKEPU (%)	CNEPU (%)	PCA (%)	PLS (%)	s-FPCA (%)	s-PCA (%)
AORD	*h* = 3	**0.07**	**1.54**	**3.42**	**0.30**	− 0.12	0.01	− 0.08	**1.02**	**1.16**	**3.05**	**3.23**
	*h* = 6	− 0.17	**0.50**	**1.01**	**0.16**	− 0.08	− 0.09	− 0.07	**0.04**	**0.20**	**1.16**	**1.00**
	*h* = 12	**0.11**	− 1.13	**0.92**	− 0.06	− 0.03	0.04	− 0.05	− 0.03	**0.44**	**0.56**	**0.61**
BFX	*h* = 3	**0.32**	**2.40**	**2.73**	− 0.29	− 0.10	**0.15**	0.14	**0.87**	**1.02**	**2.05**	**1.81**
	*h* = 6	**0.44**	**1.48**	**1.23**	0.00	− 0.10	− 0.12	− 0.15	**0.49**	**1.08**	**1.21**	**1.15**
	*h* = 12	**0.18**	**0.32**	**0.53**	0.00	− 0.06	0.01	− 0.10	− 0.03	**0.61**	**0.49**	**0.46**
BSESN	*h* = 3	**0.37**	**2.77**	**1.47**	**0.04**	− 0.07	− 0.05	− 0.09	**0.53**	**1.05**	**1.20**	**1.30**
	*h* = 6	**0.49**	**2.60**	**0.35**	**0.05**	− 0.17	− 0.04	− 0.09	**0.26**	**0.85**	**0.30**	**0.38**
	*h* = 12	**0.72**	**1.99**	− 0.02	− 0.05	− 0.08	− 0.07	− 0.07	− 0.09	**0.96**	− 0.01	0.03
BVSP	*h* = 3	**0.22**	**1.51**	**0.91**	− 0.06	− 0.07	− 0.12	**0.04**	**0.27**	**0.35**	**0.54**	**0.63**
	*h* = 6	**0.38**	**1.40**	**0.36**	− 0.01	− 0.17	− 0.04	− 0.05	**0.45**	**0.78**	**0.41**	**0.38**
	*h* = 12	**0.57**	**1.35**	**0.06**	− 0.10	− 0.13	− 0.16	− 0.13	0.03	**0.73**	**0.03**	**0.07**
DJI	*h* = 3	**0.04**	**3.17**	**1.85**	− 0.03	− 0.10	− 0.06	− 0.01	**0.29**	**0.45**	**1.35**	**1.33**
	*h* = 6	**0.04**	**2.41**	**1.06**	− 0.09	− 0.15	− 0.09	− 0.13	**0.44**	**0.42**	**1.00**	**1.01**
	*h* = 12	**0.01**	**1.90**	**0.55**	− 0.09	− 0.12	0.00	− 0.10	− 0.04	**0.16**	**0.44**	**0.40**
FCHI	*h* = 3	**0.40**	**2.79**	**2.91**	− 0.35	− 0.09	**0.11**	0.05	**0.64**	**1.11**	**2.04**	**1.81**
	*h* = 6	**0.40**	**2.14**	**0.92**	**0.04**	− 0.11	− 0.11	− 0.17	**0.38**	**0.97**	**1.02**	**0.97**
	*h* = 12	**0.54**	**1.54**	**0.44**	− 0.02	− 0.10	0.05	− 0.12	**0.01**	**0.93**	**0.41**	**0.40**
FTMIB	*h* = 3	**0.18**	**1.48**	**2.88**	− 0.46	− 0.13	− 0.28	0.05	**0.38**	**0.79**	**2.61**	**2.20**
	*h* = 6	**0.40**	**0.54**	**1.15**	− 0.08	− 0.09	− 0.10	− 0.21	**0.33**	**0.88**	**1.21**	**1.25**
	*h* = 12	**0.29**	− 0.06	0.22	0.01	− 0.11	− 0.06	− 0.06	− 0.09	**0.51**	**0.25**	**0.30**
FTSE	*h* = 3	**0.24**	**3.48**	**3.09**	− 0.15	− 0.15	− 0.02	− 0.02	**0.93**	**1.07**	**2.53**	**2.57**
	*h* = 6	**0.09**	**2.25**	**0.98**	**0.08**	− 0.13	− 0.12	− 0.11	**0.37**	**0.63**	**1.11**	**1.01**
	*h* = 12	**0.24**	**1.17**	**0.75**	0.01	− 0.15	− 0.02	− 0.13	**0.09**	**0.65**	**0.66**	**0.57**
GDAXI	*h* = 3	**0.19**	**2.28**	**2.29**	− 0.29	− 0.11	0.04	0.02	**0.36**	**0.74**	**1.74**	**1.53**
	*h* = 6	**0.26**	**1.92**	**0.48**	− 0.06	− 0.08	− 0.12	− 0.13	**0.22**	**0.64**	**0.64**	**0.53**
	*h* = 12	**0.41**	**2.09**	**0.37**	0.00	− 0.11	− 0.08	− 0.05	− 0.01	**0.76**	**0.31**	**0.30**
GSPTSE	*h* = 3	0.00	**1.36**	**1.29**	− 0.16	− 0.10	− 0.12	− 0.07	**0.35**	**0.28**	**1.14**	**1.10**
	*h* = 6	− 0.11	**0.40**	**0.41**	− 0.07	− 0.13	− 0.10	− 0.08	**0.17**	**0.06**	**0.48**	**0.44**
	*h* = 12	− 0.06	− 0.36	**0.45**	− 0.08	− 0.09	− 0.09	− 0.12	− 0.13	**0.08**	**0.46**	**0.43**
HSI	*h* = 3	**0.12**	**1.53**	**1.66**	− 0.09	− 0.18	− 0.06	− 0.14	**0.73**	**0.75**	**1.34**	**1.39**
	*h* = 6	**0.07**	**1.08**	**0.49**	− 0.11	− 0.08	− 0.05	− 0.15	**0.03**	**0.34**	**0.41**	**0.49**
	*h* = 12	**0.18**	**0.40**	**0.24**	− 0.04	− 0.09	− 0.06	− 0.12	**0.12**	**0.47**	**0.32**	**0.25**
IBEX	*h* = 3	**0.36**	**1.38**	**2.05**	− 0.19	− 0.12	0.00	**0.07**	**0.72**	**0.86**	**1.56**	**1.39**
	*h* = 6	**0.44**	**1.15**	**0.64**	**0.02**	− 0.11	− 0.05	− 0.14	**0.27**	**0.83**	**0.56**	**0.55**
	*h* = 12	**0.16**	**0.27**	**0.35**	− 0.04	− 0.08	− 0.02	− 0.11	− 0.06	**0.45**	**0.16**	**0.21**
KS11	*h* = 3	**0.34**	**2.46**	**2.32**	− 0.05	− 0.12	− 0.01	− 0.06	**0.59**	**1.16**	**1.96**	**2.18**
	*h* = 6	**0.22**	**1.67**	**0.41**	0.01	− 0.14	− 0.12	− 0.20	0.00	**0.68**	**0.35**	**0.38**
	*h* = 12	**1.08**	**1.54**	**0.67**	− 0.13	− 0.11	− 0.09	− 0.16	**0.07**	**1.61**	**0.66**	**0.76**
KSE	*h* = 3	− 0.14	**0.60**	− 0.04	0.05	− 0.02	0.00	− 0.13	− 0.01	**0.24**	**0.28**	**0.26**
	*h* = 6	− 0.42	**0.47**	− 0.02	− 0.07	− 0.07	− 0.09	− 0.08	− 0.09	− 0.10	**0.04**	**0.06**
	*h* = 12	− 0.82	**0.18**	**0.13**	− 0.11	− 0.05	− 0.09	− 0.14	− 0.28	− 0.55	**0.14**	**0.19**
MXX	*h* = 3	**0.13**	**0.75**	**1.11**	− 0.06	− 0.14	− 0.10	− 0.06	**0.33**	**0.36**	**0.52**	**0.74**
	*h* = 6	− 0.07	**0.07**	**0.67**	− 0.13	− 0.02	− 0.09	− 0.05	**0.31**	**0.17**	**0.71**	**0.74**
	*h* = 12	**0.01**	− 0.77	**0.11**	− 0.10	− 0.10	− 0.09	− 0.18	− 0.10	**0.10**	0.04	**0.12**
N225	*h* = 3	**0.62**	**0.80**	**3.06**	**0.17**	− 0.11	0.03	− 0.07	**1.57**	**1.89**	**3.08**	**3.14**
	*h* = 6	**0.29**	**0.21**	**1.16**	− 0.05	− 0.14	− 0.04	− 0.12	**0.43**	**0.80**	**1.24**	**1.36**
	*h* = 12	− 0.07	− 0.39	**0.68**	0.00	0.04	− 0.12	− 0.12	**0.18**	**0.29**	**0.74**	**0.65**
OMXC20	*h* = 3	**0.17**	**0.97**	**1.24**	− 0.04	− 0.15	− 0.04	0.00	**0.54**	**0.74**	**1.20**	**0.86**
	*h* = 6	**0.13**	**0.35**	0.02	− 0.11	− 0.04	− 0.16	− 0.17	− 0.02	**0.30**	**0.02**	− 0.01
	*h* = 12	**0.09**	− 0.28	**0.53**	− 0.05	− 0.02	− 0.11	− 0.12	− 0.11	**0.34**	**0.52**	**0.47**
OMXHPI	*h* = 3	**0.38**	**3.40**	**2.89**	− 0.43	− 0.02	− 0.07	− 0.07	**1.03**	**1.09**	**2.88**	**2.68**
	*h* = 6	**0.47**	**2.48**	**0.79**	**0.07**	− 0.03	− 0.19	− 0.06	**0.71**	**1.22**	**0.86**	**0.99**
	*h* = 12	**0.17**	**1.23**	**0.72**	− 0.01	− 0.12	− 0.02	− 0.08	**0.24**	**0.59**	**0.73**	**0.73**
OMXSPI	*h* = 3	**0.30**	**2.43**	**3.15**	− 0.30	− 0.04	− 0.01	− 0.11	**1.36**	**1.29**	**3.23**	**2.90**
	*h* = 6	**0.33**	**1.42**	**0.87**	− 0.29	− 0.04	− 0.09	− 0.05	**0.26**	**0.74**	**0.94**	**1.02**
	*h* = 12	**0.18**	**0.48**	**0.75**	− 0.04	− 0.13	− 0.05	− 0.11	**0.23**	**0.60**	**0.72**	**0.71**
OSEAX	*h* = 3	**0.22**	**2.78**	**3.16**	− 0.05	− 0.13	− 0.06	− 0.11	**1.16**	**1.23**	**2.89**	**2.93**
	*h* = 6	− 0.13	**1.32**	**0.97**	− 0.09	− 0.09	− 0.08	− 0.16	**0.30**	**0.44**	**0.86**	**0.81**
	*h* = 12	**0.05**	**0.15**	**0.73**	− 0.05	− 0.13	− 0.06	− 0.07	**0.09**	**0.42**	**0.51**	**0.49**
SSEC	*h* = 3	− 0.09	− 0.46	**0.19**	− 0.07	− 0.16	− 0.11	− 0.06	− 0.08	− 0.07	**0.09**	**0.05**
	*h* = 6	− 0.07	− 0.49	− 0.09	− 0.05	− 0.06	− 0.07	− 0.10	− 0.02	− 0.08	0.00	− 0.12
	*h* = 12	− 0.05	− 0.38	− 0.08	− 0.08	− 0.08	− 0.11	− 0.09	− 0.11	0.02	− 0.08	− 0.08
SSMI	*h* = 3	**0.10**	**1.39**	**2.78**	− 0.18	− 0.08	− 0.02	− 0.01	**0.80**	**0.74**	**2.28**	**2.36**
	*h* = 6	**0.10**	**0.55**	**0.92**	0.01	− 0.11	− 0.14	− 0.09	**0.29**	**0.64**	**0.77**	**0.69**
	*h* = 12	**0.07**	− 0.41	**0.63**	− 0.06	− 0.05	− 0.07	− 0.09	− 0.09	**0.31**	**0.43**	**0.46**
STI	*h* = 3	**0.40**	**5.40**	**1.40**	− 0.10	− 0.10	− 0.10	0.10	**0.30**	**1.10**	**1.10**	**1.50**
	*h* = 6	**0.20**	**6.50**	**0.70**	− 0.10	− 0.20	0.00	− 0.10	0.001	**0.70**	**0.80**	**0.90**
	*h* = 12	**0.60**	**5.00**	**0.60**	0.00	0.00	0.00	− 0.10	**0.40**	**1.30**	**0.60**	**0.70**
STOXX50E	*h* = 3	**0.33**	**3.35**	**2.70**	− 0.14	− 0.15	**0.11**	0.01	**1.15**	**1.11**	**2.00**	**1.85**
	*h* = 6	**0.34**	**2.73**	**0.95**	0.00	− 0.11	− 0.10	− 0.14	**0.28**	**0.84**	**0.90**	**0.75**
	*h* = 12	**0.54**	**2.43**	**0.20**	− 0.05	− 0.11	0.04	− 0.23	− 0.05	**0.81**	0.05	0.07

This table reports out-of-sample results for multi-step-ahead forecast based on models (7) and (8). *h* donates the forecast horizon that takes the values of 3, 6, and 12. The bold font highlights the significantly positive $R_{\mathrm {OS}}^{2}$s based on the Clark and West (2007) test, and the underline font highlights the biggest one

### Robustness analyses

#### Robustness check for different window lengths

Table 7 presents the out-of-sample results when the lengths of the rolling window (*W*) were set at 2000 and 3000. We observed that the changes in the window lengths exerted weak impacts on the results reported above. VIX and DVIX were also the most significant single uncertainty indicators for international stock markets. Particularly, DVIX exerted a significant predictive power on RVs of all the markets, including KSE, where it performed poorly when *W*=1000. Moreover, PLS could not predict the stock volatility in Finland (OMXHPI) and Sweden (OMXSPI) when *W*=3000, indicating that its predictive power was unstable in several cases. Finally, s-PCA exhibited more robust and outstanding predictabilities in the composite indexes. Overall, the results were robust when the window lengths were changed in the rolling regression framework.

**Table 7:** Robustness check for different windows

Asset	Window	EMU (%)	VIX (%)	DVIX (%)	VOL (%)	USEPU (%)	UKEPU (%)	CNEPU (%)	PCA (%)	PLS (%)	s-FPCA (%)	s-PCA (%)
AORD	*W* = 2000	**0.72**	**4.87**	**6.25**	**2.50**	0.06	**0.11**	− 0.02	**5.27**	**4.04**	**6.08**	**6.44**
	*W* = 3000	**0.48**	**4.98**	**6.53**	**2.18**	0.10	0.10	− 0.08	**6.01**	**2.91**	**6.85**	**6.98**
BFX	*W* = 2000	**0.09**	**3.41**	**6.93**	**0.17**	− 0.10	− 0.04	− 0.07	**2.49**	**1.61**	**6.37**	**6.23**
	*W* = 3000	− 0.09	**4.12**	**7.39**	**0.31**	− 0.10	− 0.08	− 0.06	**3.24**	**0.80**	**7.50**	**7.53**
BSESN	*W* = 2000	**0.28**	**2.40**	**2.30**	**0.08**	0.01	− 0.10	− 0.11	**0.96**	**1.08**	**2.21**	**2.42**
	*W* = 3000	**0.17**	**2.29**	**2.84**	− 0.14	− 0.16	0.02	− 0.02	**1.00**	**0.75**	**2.87**	**2.80**
BVSP	*W* = 2000	**0.33**	**0.80**	**1.80**	**0.08**	− 0.09	− 0.06	− 0.09	**1.62**	**1.04**	**1.71**	**1.68**
	*W* = 3000	**0.31**	**1.77**	**1.40**	**0.27**	− 0.05	− 0.05	− 0.31	**1.52**	**0.95**	**1.49**	**1.55**
DJI	*W* = 2000	**0.51**	**1.78**	**4.45**	− 0.07	0.01	0.03	− 0.09	**1.35**	**1.34**	**3.83**	**3.36**
	*W* = 3000	**0.30**	**3.53**	**4.70**	− 0.04	− 0.07	0.01	0.00	**1.73**	**0.91**	**4.69**	**4.17**
FCHI	*W* = 2000	− 0.03	**2.05**	**6.52**	**0.16**	− 0.09	− 0.04	− 0.13	**2.09**	**1.35**	**6.21**	**5.83**
	*W* = 3000	− 0.37	**2.44**	**6.80**	**0.61**	− 0.10	− 0.05	− 0.10	**2.88**	**0.23**	**6.91**	**6.76**
FTMIB	*W* = 2000	− 0.63	− 1.78	**6.37**	**0.78**	0.08	− 0.22	− 0.10	**0.68**	**0.19**	**6.15**	**6.09**
	*W* = 3000	–	–	–	–	–	–	–	–	–	–	–
FTSE	*W* = 2000	**0.22**	**5.06**	**5.61**	**0.23**	0.09	− 0.03	− 0.09	**3.42**	**2.16**	**5.09**	**4.86**
	*W* = 3000	0.08	**6.39**	**5.28**	**1.36**	0.00	− 0.02	− 0.03	**4.09**	**1.41**	**5.55**	**5.64**
GDAXI	*W* = 2000	− 0.17	**1.31**	**6.12**	**0.30**	0.02	− 0.06	− 0.08	**2.62**	**1.05**	**5.65**	**5.31**
	*W* = 3000	− 0.28	**1.93**	**5.93**	**1.09**	0.05	− 0.04	− 0.05	**3.28**	**0.15**	**6.08**	**6.19**
GSPTSE	*W* = 2000	− 0.05	**1.49**	**3.15**	0.02	− 0.01	− 0.04	− 0.16	**1.39**	**0.87**	**2.92**	**2.90**
	*W* = 3000	− 0.21	**1.21**	**4.46**	**0.36**	0.03	− 0.02	− 0.12	**2.24**	**0.41**	**4.58**	**4.53**
HSI	*W* = 2000	**0.64**	**3.01**	**2.31**	**0.08**	− 0.01	0.01	− 0.25	**1.47**	**1.55**	**2.48**	**2.30**
	*W* = 3000	**0.23**	**3.14**	**2.01**	− 0.06	− 0.02	− 0.07	− 0.14	**1.08**	**0.70**	**2.07**	**2.36**
IBEX	*W* = 2000	− 0.05	**0.08**	**4.36**	**0.12**	− 0.13	− 0.03	− 0.11	**1.65**	**0.98**	**4.26**	**3.96**
	*W* = 3000	− 0.29	**1.39**	**3.40**	**0.46**	− 0.18	− 0.04	− 0.04	**1.99**	**0.11**	**3.50**	**3.51**
KS11	*W* = 2000	**0.60**	**2.75**	**4.79**	**0.28**	− 0.01	0.03	− 0.08	**2.69**	**2.02**	**4.58**	**4.58**
	*W* = 3000	**0.32**	**3.02**	**5.24**	**0.23**	− 0.04	− 0.02	0.02	**2.30**	**1.36**	**5.33**	**5.61**
KSE	*W* = 2000	**0.37**	**0.80**	**0.15**	− 0.13	− 0.10	− 0.20	− 0.10	**0.17**	**0.48**	**0.10**	**0.07**
	*W* = 3000	**0.36**	**0.88**	**0.25**	− 0.05	− 0.03	**0.03**	− 0.08	**0.34**	**0.48**	**0.28**	**0.33**
MXX	*W* = 2000	**0.70**	**2.43**	**1.13**	− 0.12	− 0.06	− 0.02	0.00	**0.65**	**1.08**	**1.12**	**0.82**
	*W* = 3000	**0.47**	**2.19**	**0.81**	**0.20**	0.04	0.00	− 0.04	**0.89**	**0.88**	**0.87**	**1.02**
N225	*W* = 2000	**0.56**	**1.71**	**6.53**	**0.87**	− 0.04	− 0.10	− 0.06	**3.38**	**2.69**	**6.55**	**7.03**
	*W* = 3000	0.00	**3.17**	**7.15**	**0.95**	− 0.04	− 0.07	**0.02**	**4.02**	**1.63**	**7.33**	**7.76**
OMXC20	*W* = 2000	− 0.19	**2.44**	**3.08**	**1.40**	0.00	− 0.08	− 0.12	**2.73**	**1.55**	**2.80**	**3.09**
	*W* = 3000	− 0.35	**2.22**	**1.51**	**1.79**	− 0.10	− 0.16	− 0.09	**1.23**	**0.06**	**1.70**	**1.85**
OMXHPI	*W* = 2000	− 0.56	**3.17**	**6.62**	**1.58**	− 0.23	− 0.08	− 0.13	**4.57**	**1.65**	**6.70**	**6.49**
	*W* = 3000	− 1.20	**2.32**	**6.03**	**1.53**	0.05	− 0.02	− 0.32	**2.64**	− 1.10	**6.24**	**6.24**
OMXSPI	*W* = 2000	− 0.28	**3.46**	**8.08**	**1.05**	0.07	0.00	− 0.08	**4.86**	**1.94**	**7.53**	**7.32**
	*W* = 3000	− 0.63	**3.13**	**8.41**	**1.83**	**0.30**	− 0.11	− 0.01	**4.36**	− 0.18	**8.67**	**8.58**
OSEAX	*W* = 2000	**0.21**	**3.44**	**4.95**	**0.60**	0.06	− 0.05	− 0.09	**2.96**	**1.92**	**4.44**	**4.31**
	*W* = 3000	− 0.15	**5.48**	**4.42**	**0.92**	0.01	− 0.05	− 0.09	**3.08**	**0.75**	**4.57**	**4.69**
SSEC	*W* = 2000	− 0.05	0.05	**2.19**	**0.07**	− 0.06	− 0.06	− 0.13	**0.71**	**0.16**	**2.20**	**2.35**
	*W* = 3000	− 0.04	0.09	**2.54**	0.06	− 0.08	− 0.12	− 0.22	**1.23**	**0.09**	**2.58**	**2.63**
SSMI	*W* = 2000	**0.22**	**2.67**	**5.85**	**0.48**	0.07	− 0.06	− 0.03	**2.74**	**1.81**	**5.31**	**5.20**
	*W* = 3000	− 0.12	**3.43**	**5.36**	**0.90**	0.07	− 0.03	− 0.01	**3.23**	**0.71**	**5.49**	**5.37**
STOXX50E	*W* = 2000	**0.04**	**1.42**	**4.86**	− 0.26	− 0.23	− 0.01	− 0.04	**1.97**	**1.27**	**4.85**	**4.66**
	*W* = 3000	− 0.21	**2.26**	**4.77**	**0.63**	− 0.14	− 0.03	− 0.04	**2.50**	**0.37**	**4.90**	**4.74**

This table reports out-of-sample results for robustness check using different window lengths (*W*) in the rolling regression framework based on models (7) and (8). The bold font highlights the significantly positive $R_{\mathrm {OS}}^{2}$s based on the Clark and West (2007) test, and the underline font highlights the biggest one

#### Robustness check for the business cycle

The predictability of stock volatility has been proven to change over time. Paye (2012) observed that the predictive performance changed in different subperiods. This subsection discussed a robustness check to identify whether the out-of-sample predictability changed in the business cycle. Table 8 presents the out-of-sample results during the NBER-dated U.S. economic expansions and contractions.

Regarding the single UI, we observed that DVIX exhibited robust predictive ability during the economic expansions and recessions in most markets except for KSE and Mexico (MXX). Moreover, VIX exhibited poor performance during economic recessions in many countries, including Belgium (BFX), America (DJI), the U.K. (FTSE), Spain (IBEX), Japan (N225), Denmark (OMXC20), Sweden (OMXSPI), Norway (OSEAX), China (SSEC), and Switzerland (SSMI). This indicated that VIX was not a robust predictor in many markets, which the extant literature did not report, e.g., Wang et al. (2020a) and Liang et al. (2020). Further, this result highlights that DVIX was superior to VIX regarding robustness. Moreover, EMU and VOL exerted robust explanatory powers on potential RVs during expansions and recessions in most stock markets, indicating that they were relatively significant volatility predictors for forecasting international stock market volatilities. Finally, EPUs performed poorly in both periods, as always.

Regarding the composite UIs, dissimilar to VIX, PCA exhibited a weak predictive ability over the economic contractions in a few countries. This result is consistent with that of Gong et al. (2022) who observed that the investor sentiment predicted stock volatility better under economic expansion conditions than under recession ones. This might be related to the increases in uncertainty during an economic recession, which results in poor predictive performance employing an unsupervised learning method, such as PCA. Moreover, PLS and s-PCA were the only robust indexes that exerted a significant predictive power in both expansions and recessions based on the positive $R_{\mathrm {OS}}^{2}$. Interestingly, for PLS, we observed that it exhibited a better out-of-sample performance during recessions than during expansions, indicating that the PLS method could capture more prediction information during economic recessions.

**Table 8:** Robustness check for the business cycle

Asset	Cycle	EMU (%)	VIX (%)	DVIX (%)	VOL (%)	USEPU (%)	UKEPU (%)	CNEPU (%)	PCA (%)	PLS (%)	s-FPCA (%)	s-PCA (%)
AORD	Exp.	**0.20**	**2.34**	**5.59**	**3.12**	**0.12**	− 0.07	− 0.04	**3.31**	**3.48**	**6.88**	**7.47**
	Rec.	**2.84**	**5.75**	**8.90**	**1.25**	0.06	− 0.05	− 0.28	**2.13**	**11.30**	**8.89**	**7.32**
BFX	Exp.	**0.17**	**4.99**	**6.80**	**0.37**	− 0.08	**0.02**	− 0.10	**2.59**	**1.74**	**6.09**	**6.17**
	Rec.	− 0.01	− 1.15	**8.51**	**0.36**	− 0.21	0.14	0.16	**1.97**	**4.02**	**3.97**	**3.66**
BSESN	Exp.	**0.18**	**0.64**	**1.72**	**0.32**	0.00	− 0.14	− 0.15	**0.99**	**0.99**	**1.84**	**1.88**
	Rec.	**2.49**	**14.75**	**5.22**	− 0.01	− 0.38	− 0.09	− 0.01	**2.35**	**5.21**	**5.24**	**6.12**
BVSP	Exp.	**0.14**	**0.80**	**1.61**	**0.06**	− 0.12	− 0.12	− 0.05	**1.09**	**0.72**	**1.55**	**1.77**
	Rec.	**0.48**	**3.49**	**1.26**	0.00	0.06	− 0.01	0.22	**1.05**	**2.12**	**2.17**	**2.19**
DJI	Exp.	**0.40**	**7.88**	**5.03**	− 0.11	− 0.08	− 0.03	− 0.11	**1.47**	**1.23**	**4.03**	**3.87**
	Rec.	**0.60**	− 20.65	**3.17**	− 0.06	0.14	0.05	0.01	**1.48**	**1.74**	**0.05**	**2.27**
FCHI	Exp.	**0.29**	**4.38**	**7.25**	**0.82**	− 0.03	− 0.10	− 0.12	**2.49**	**1.90**	**6.33**	**6.45**
	Rec.	− 0.29	**3.45**	**8.52**	**0.41**	− 0.43	− 0.01	0.18	**2.21**	**5.04**	**4.98**	**4.69**
FTMIB	Exp.	− 0.06	**2.61**	**5.99**	**0.34**	− 0.04	− 0.05	− 0.15	**2.16**	**1.03**	**6.12**	**5.86**
	Rec.	− 3.56	**3.66**	**12.47**	**1.13**	0.07	− 0.14	− 1.13	− 2.26	**7.55**	2.39	0.19
FTSE	Exp.	**0.07**	**6.63**	**5.24**	**1.33**	− 0.03	− 0.07	− 0.09	**3.00**	**2.35**	**6.11**	**6.56**
	Rec.	**0.61**	− 4.90	**5.05**	**0.53**	− 0.16	− 0.12	− 0.32	**0.56**	**5.39**	**4.09**	**2.97**
GDAXI	Exp.	**0.10**	**2.28**	**6.02**	**1.06**	− 0.18	− 0.09	− 0.17	**2.46**	**1.43**	**5.95**	**6.08**
	Rec.	− 0.63	**9.40**	**7.50**	**0.49**	− 0.28	− 0.02	0.03	**1.80**	**5.01**	**5.10**	**2.97**
GSPTSE	Exp.	− 0.05	**2.53**	**3.18**	**0.15**	− 0.09	− 0.14	− 0.18	**1.28**	**0.74**	**3.09**	**3.06**
	Rec.	− 0.04	**3.89**	**2.22**	− 0.55	**0.69**	− 0.10	0.11	**1.25**	**1.47**	**1.58**	**2.24**
HSI	Exp.	**0.29**	**2.24**	**2.32**	**0.26**	− 0.09	− 0.04	− 0.24	**1.27**	**1.25**	**2.35**	**2.66**
	Rec.	**1.32**	**0.15**	**7.25**	**0.46**	− 0.05	− 0.24	0.25	**1.99**	**4.88**	**3.72**	**3.91**
IBEX	Exp.	**0.35**	**2.42**	**5.03**	**0.42**	− 0.06	− 0.05	− 0.04	**2.17**	**1.42**	**4.34**	**4.85**
	Rec.	− 0.32	− 1.35	**8.67**	0.16	− 0.11	0.10	− 0.29	**3.65**	**3.86**	**4.96**	**4.63**
KS11	Exp.	**0.45**	**2.19**	**4.58**	**0.28**	− 0.19	− 0.17	− 0.21	**2.15**	**1.92**	**4.00**	**4.43**
	Rec.	**1.40**	**12.21**	**8.91**	− 0.71	0.04	− 0.01	− 0.05	− 0.34	**4.23**	**3.39**	**5.85**
KSE	Exp.	− 0.12	**0.10**	− 0.01	− 0.18	− 0.05	− 0.18	− 0.14	− 0.04	**0.06**	**0.06**	**0.07**
	Rec.	0.39	**1.27**	− 0.11	− 0.19	0.24	− 0.55	− 0.03	0.27	0.05	− 0.25	0.01
MXX	Exp.	**0.19**	**0.81**	**1.24**	− 0.02	− 0.19	− 0.10	− 0.12	**0.54**	**0.45**	**1.24**	**1.37**
	Rec.	**1.08**	**1.91**	− 0.10	− 0.18	0.02	− 0.03	**0.16**	**1.02**	**2.44**	− 0.14	0.39
N225	Exp.	**0.38**	**2.44**	**6.13**	**0.73**	− 0.16	− 0.03	− 0.13	**3.10**	**2.54**	**6.27**	**6.66**
	Rec.	**2.06**	− 5.67	**15.56**	**1.90**	− 0.02	− 0.03	− 0.14	**1.59**	**10.36**	**17.91**	**18.24**
OMXC20	Exp.	**0.55**	**3.07**	**3.10**	**0.76**	− 0.15	− 0.02	− 0.11	**2.69**	**2.29**	**3.40**	**3.68**
	Rec.	**1.60**	− 22.67	7.99	**2.30**	0.42	− 0.21	− 0.07	**1.96**	**11.10**	9.30	**12.07**
OMXHPI	Exp.	**0.70**	**5.29**	**5.76**	**0.65**	− 0.21	− 0.05	− 0.05	**4.68**	**2.78**	**5.98**	**6.12**
	Rec.	0.41	**9.18**	**9.70**	**2.80**	− 0.15	0.09	0.14	− 1.03	**10.43**	**10.86**	**13.20**
OMXSPI	Exp.	**0.47**	**5.55**	**7.48**	**0.34**	− 0.24	**0.01**	− 0.11	**4.88**	**2.44**	**7.47**	**7.50**
	Rec.	**1.12**	− 11.05	**17.72**	**3.26**	0.12	0.03	0.44	− 0.47	**14.13**	**19.32**	**22.36**
OSEAX	Exp.	**0.13**	**5.74**	**4.42**	**0.74**	− 0.18	− 0.08	− 0.11	**3.11**	**2.00**	**4.80**	**5.09**
	Rec.	0.06	− 3.22	**6.88**	**2.33**	− 0.14	0.00	0.01	**3.90**	**6.29**	**4.49**	**3.80**
SSEC	Exp.	− 0.07	**0.09**	**1.74**	− 0.03	− 0.11	− 0.03	− 0.17	**0.63**	**0.16**	**1.54**	**1.42**
	Rec.	− 0.28	− 2.51	**5.82**	− 0.26	− 0.10	0.48	0.02	**0.09**	**1.46**	**3.01**	**2.90**
SSMI	Exp.	**0.23**	**3.24**	**6.81**	**0.77**	0.00	− 0.11	− 0.02	**2.88**	**1.90**	**5.88**	**6.34**
	Rec.	**0.38**	− 0.63	**7.62**	**1.48**	− 0.11	− 0.02	0.20	**2.46**	**4.23**	**4.01**	**3.56**
STOXX50E	Exp.	**0.15**	**3.83**	**5.24**	**1.42**	− 0.12	− 0.02	− 0.14	**2.67**	**1.96**	**5.80**	**5.89**
	Rec.	0.04	**8.93**	**6.58**	**0.13**	− 0.62	− 0.10	− 0.17	**2.42**	**6.09**	**2.64**	**1.96**

This table reports out-of-sample results during the NBER-dated economic expansions (Exp.) and recessions (Rec.) based on models (7) and (8). The bold font highlights the significantly positive $R_{\mathrm {OS}}^{2}$s based on the Clark and West (2007) test, and the underline font highlights the biggest one

#### Robustness check employing realized semi-variances as the response variable

Although RV, which has attracted enormous attention in the literature, is a popular measure for identifying market risks, the realized semi-variance, which captures the impacts of negative returns (downside risk), could be more relevant to investors. This measure was developed by Barndorff-Nielsen et al. (2010) and defined by the following equation:

16 $$\begin{aligned} R S_{t}=\sum _{j=1}^{M_{t}}I_{r_{t,j}<0}\cdot r_{t, j}^{2}, \end{aligned}$$

where $I_{r_{t,j}<0}$ is an indicator function that takes the value of unity if $r_{t,j}<0$ and zero otherwise. We replaced (log)RV with (log)RS in the regression models (7) and (8). Table 9 reports the results of whether UIs impacted the realized semi-variance in global stock markets. The results demonstrated that the findings were consistent with RV. More specifically, VIX, DVIX, and s-PCA were the main, significant, and powerful contributors to the prediction of stock downside risks in international markets, respectively. Moreover, some UIs exerted a significantly higher predictive power on the Australian stock market, as evidenced by the large $R_{\mathrm {OS}}^{2}$s (27.25% and 22.99% for DVIX and s-PCA, respectively).

**Table 9:** Robustness check for using realized semi-variance as dependent variable

Asset	EMU	VIX	DVIX	VOL	USEPU	UKEPU	CNEPU	PCA	PLS	s-FPCA	s-PCA
AORD	**0.00%**	**3.15%**	**27.25%**	**1.97%**	− 0.02%	− 0.06%	− 0.07%	**7.93%**	**5.32%**	**22.57%**	**22.99%**
	(1.945*)	(9.021***)	(19.287***)	(8.688***)	(0.779)	(0.151)	(0.526)	(14.187***)	(11.494***)	(17.559***)	(16.315***)
BFX	− 0.02%	**3.03%**	**3.61%**	**0.34%**	− 0.11%	**0.11%**	− 0.13%	**1.32%**	**1.14%**	**3.13%**	**3.13%**
	(1.220)	(8.414***)	(8.920***)	(3.760***)	(− 0.285)	(2.229**)	(− 2.180**)	(6.615***)	(6.164***)	(8.088***)	(7.637***)
BSESN	**0.31%**	**1.80%**	**1.52%**	**0.04%**	− 0.04%	− 0.11%	− 0.09%	**0.76%**	**1.01%**	**1.16%**	**1.37%**
	(3.403***)	(5.905***)	(7.153***)	(1.695*)	(0.893)	(0.224)	(0.907)	(5.675***)	(5.729***)	(6.537***)	(6.797***)
BVSP	**0.05%**	**0.69%**	**0.31%**	− 0.02%	− 0.14%	− 0.11%	− 0.09%	**0.42%**	**0.40%**	**0.46%**	**0.56%**
	(2.102**)	(5.015***)	(3.571***)	(1.281)	(− 0.723)	(− 1.130)	(− 0.180)	(4.073***)	(4.074***)	(4.236***)	(4.710***)
DJI	**0.09%**	**1.80%**	**0.36%**	− 0.11%	− 0.11%	− 0.10%	− 0.16%	**0.15%**	**0.32%**	**0.26%**	**0.36%**
	(1.851*)	(8.427***)	(3.480***)	(− 0.607)	(− 1.001)	(− 2.816**)	(− 0.777)	(2.289**)	(3.309***)	(2.864***)	(3.231***)
FCHI	**0.08%**	**2.96%**	**3.76%**	**0.73%**	− 0.11%	− 0.05%	− 0.12%	**1.32%**	**1.45%**	**3.50%**	**3.55%**
	(2.133**)	(8.943***)	(9.869***)	(5.374***)	(− 0.390)	(0.217)	(− 1.476)	(6.938***)	(7.150***)	(9.621***)	(8.958***)
FTMIB	− 0.16%	**1.70%**	**2.80%**	**0.35%**	− 0.10%	− 0.02%	− 0.20%	**0.91%**	**0.77%**	**2.73%**	**2.78%**
	(0.528)	(5.177***)	(6.628***)	(2.827***)	(− 0.122)	(0.469)	(− 1.320)	(3.853***)	(3.611***)	(6.311***)	(5.834***)
FTSE	**0.15%**	**4.21%**	**9.36%**	**0.89%**	− 0.08%	0.00%	− 0.13%	**3.55%**	**2.85%**	**7.41%**	**7.97%**
	(2.539**)	(9.941***)	(13.069***)	(5.894***)	(0.530)	(0.994)	(− 0.396)	(9.919***)	(9.515***)	(11.500***)	(10.304***)
GDAXI	− 0.01%	**1.99%**	**3.49%**	**0.74%**	− 0.18%	− 0.03%	− 0.18%	**1.36%**	**1.12%**	**2.99%**	**3.22%**
	(1.570)	(8.347***)	(9.522***)	(4.950***)	(− 0.884)	(0.737)	(− 1.777*)	(6.808***)	(6.243***)	(9.438***)	(9.281***)
GSPTSE	− 0.10%	**1.33%**	**1.06%**	− 0.03%	− 0.14%	− 0.04%	− 0.06%	**0.63%**	**0.33%**	**0.87%**	**1.01%**
	(0.453)	(6.703***)	(5.398***)	(1.197)	(− 1.413)	(0.623)	(0.196)	(4.370***)	(3.336***)	(4.870***)	(5.142***)
HSI	**0.29%**	**1.41%**	**0.37%**	− 0.06%	− 0.15%	− 0.12%	− 0.19%	**0.31%**	**0.75%**	**0.38%**	**0.51%**
	(3.328***)	(6.599***)	(3.658***)	(1.164)	(− 2.253**)	(− 1.029)	(− 0.109)	(3.185***)	(4.896***)	(3.636***)	(3.655***)
IBEX	**0.08%**	**1.57%**	**2.31%**	**0.47%**	− 0.11%	**0.08%**	− 0.06%	**1.06%**	**1.04%**	**2.12%**	**2.35%**
	(2.053**)	(7.815***)	(7.922***)	(4.280***)	(0.547)	(1.777*)	(0.361)	(6.291***)	(6.232***)	(7.892***)	(7.740***)
KS11	**0.48%**	**2.31%**	**1.13%**	− 0.09%	− 0.14%	− 0.23%	− 0.15%	**0.34%**	**1.02%**	**0.66%**	**1.01%**
	(4.145***)	(7.185***)	(5.891***)	(− 0.027)	(− 0.993)	(− 1.042)	(− 1.028)	(3.507***)	(5.780***)	(4.443***)	(5.104***)
KSE	− 0.11%	**0.17%**	**0.43%**	− 0.17%	− 0.01%	− 0.13%	− 0.10%	**0.08%**	0.02%	**0.37%**	**0.35%**
	(0.299)	(2.463**)	(4.235***)	(− 0.898)	(0.848)	(− 0.164)	(− 1.463)	(2.068**)	(1.586)	(3.965***)	(3.979***)
MXX	**0.12%**	**0.17%**	**0.16%**	− 0.09%	− 0.27%	− 0.11%	− 0.08%	**0.15%**	**0.25%**	**0.14%**	**0.21%**
	(2.372**)	(4.353***)	(2.639***)	(1.091)	(− 2.857***)	(− 1.716*)	(− 0.948)	(2.362**)	(3.220***)	(2.761***)	(2.839***)
N225	**0.25%**	**1.15%**	**5.86%**	**0.51%**	− 0.14%	− 0.12%	− 0.13%	**2.57%**	**2.12%**	**5.97%**	**6.39%**
	(3.250***)	(6.506***)	(10.649***)	(4.254***)	(− 3.016***)	(− 1.680*)	(− 0.761)	(7.849***)	(7.525***)	(10.285***)	(9.296***)
OMXC20	**0.46%**	**2.33%**	**2.44%**	**0.60%**	− 0.16%	− 0.01%	− 0.11%	**1.64%**	**2.57%**	**2.78%**	**3.15%**
	(3.852***)	(6.787***)	(6.620***)	(4.660***)	(− 1.208)	(0.822)	(− 1.413)	(6.389***)	(7.665***)	(6.646***)	(6.207***)
OMXHPI	**0.39%**	**3.86%**	**2.58%**	**0.66%**	− 0.22%	0.01%	− 0.08%	**2.71%**	**2.08%**	**2.75%**	**3.09%**
	(3.625***)	(7.915***)	(7.852***)	(3.962***)	(− 0.660)	(1.325)	(0.174)	(8.199***)	(7.253***)	(7.879***)	(7.850***)
OMXSPI	**0.24%**	**3.27%**	**4.17%**	**0.77%**	− 0.23%	**0.24%**	− 0.07%	**3.25%**	**1.88%**	**4.23%**	**4.44%**
	(2.779***)	(7.481***)	(9.330***)	(4.425***)	(− 0.480)	(2.748***)	(0.141)	(8.312***)	(6.793***)	(9.146***)	(8.867***)
OSEAX	**0.24%**	**3.17%**	**11.19%**	**0.83%**	− 0.17%	− 0.07%	− 0.12%	**5.19%**	**3.49%**	**8.58%**	**9.16%**
	(3.444***)	(9.081***)	(13.135***)	(5.459***)	(− 1.030)	(0.502)	(− 0.842)	(10.977***)	(10.051***)	(11.224***)	(10.196***)
SSEC	− 0.09%	− 0.13%	**0.46%**	− 0.09%	− 0.19%	− 0.01%	− 0.16%	**0.15%**	0.01%	**0.12%**	**0.13%**
	(0.242)	(2.485**)	(4.110***)	(− 0.600)	(− 1.547)	(0.674)	(− 0.589)	(2.132**)	(1.198)	(2.778***)	(2.804***)
SSMI	**0.17%**	**1.83%**	**3.63%**	**0.50%**	− 0.08%	− 0.09%	− 0.01%	**1.55%**	**1.52%**	**3.36%**	**3.67%**
	(2.719***)	(8.146***)	(9.403***)	(4.267***)	(− 1.480)	(− 0.436)	(0.776)	(7.469***)	(7.048***)	(9.157***)	(8.843***)
STOXX50E	− 0.01%	**2.80%**	**5.83%**	**0.81%**	− 0.18%	− 0.02%	− 0.19%	**2.15%**	**1.63%**	**4.30%**	**4.87%**
	(1.496)	(8.727***)	(11.030***)	(5.237***)	(− 0.182)	(0.289)	(− 2.850***)	(8.561***)	(7.647***)	(10.392***)	(10.007***)

This table reports out-of-sample results for using realized semi-variance in models (7) and (8). The bold font highlights the significantly positive $R_{\mathrm {OS}}^{2}$s according to the Clark and West (2007) test, and the underline font highlights the biggest one. The statistics of Clark and West (2007) test are shown in parentheses, which corresponds to test $H_0$: $R_{OS}^2\le 0$ against $H_A$: $R_{OS}^2$>0. *, ** and *** refer to statistical significance at 10%, 5% and 1% level, respectively

## Predictability analyses

The empirical results revealed significant differences among the uncertainty indicators regarding predictability. This section further analyzed the reasons. To do this, two schemes were designed. In the first one, we compared the prediction errors of all the models, and in the second, we investigated why the composite indexes delivered different out-of-sample performances by analyzing the loadings of the dimension-reduction methods.

### Comparison of the prediction error

We conducted the analyses from the following two dimensions. On the one hand, we focused on the *time dimension*, and on the other, we compared which uncertainty measure exhibited better-fitted values in longer periods. For example, if DVIX produced a smaller prediction error in more periods than the other indexes, it was considered to demonstrate a greater possibility for achieving high prediction accuracy. Conversely, we focused on the *stability dimension*. More specifically, we focused on the volatility of the prediction errors. If the residuals fluctuated wildly, it must be unstable. Many extremely predicted values (colossal prediction error) could significantly affect the prediction accuracy. Thus, we expected more stable prediction results, which exhibited less extreme predicted values.

Owing to the outstanding out-of-sample performance of DVIX, we set it as the benchmark and compared the prediction errors between it and the other UIs (*u*) over time. We first discussed the time dimension. To do this, we defined the following:

17 $$\begin{aligned} D_{t}^{u}= {\left\{ \begin{array}{ll} 1, &{} \text{ if } \left| R V_{f, t}^{\mathrm {DVIX}}-R V_{r, t}\right| \le \left| R V_{f, t}^{u}-R V_{r, t}\right| , t=1, 2, \cdots , T_{\mathrm {OS}} \\ 0. &{} \text{ otherwise } \end{array}\right. }, \end{aligned}$$

Next, we defined a “superior probability”, as follows:

18 $$\begin{aligned} p_{sup}=\frac{\sum _{t=1}^{T_{\mathrm {OS}}} D_{t}^{u}}{T_{\mathrm {OS}}}. \end{aligned}$$

The condition $\left| R V_{f, t}^{\mathrm {DVIX}}-R V_{r, t}\right| \le \left| R V_{f, t}^{u}-R V_{r, t}\right|$ indicated whether the residual error derived from the HAR-RV-DVIX model was not larger than that derived from the HAR-RV-*u* model on day *t*, where $u\in \mathrm {UI}\setminus \{\mathrm {DVIX}\}$. Thus, Eq. (18) measures the probability of DVIX to produce a smaller residual error compared with the other UIs.

Table 10 presents the superior probability, $p_{sup}$, in each market, where the bold font highlights that the probability was <50%. DVIX outperformed the other uncertainty indicators in predicting RVs during more than half of the out-of-sample periods. This is a universal phenomenon except for the s-(F)PCA indexes in most markets. Notably, the out-of-sample size was between 1994 and 4176, indicating that 1% in $p_{sup}$ denoted 20-42 observations. Thus, DVIX exhibited better performance than the others except for the s-(F)PCA indexes since it had smaller prediction errors in longer periods.

**Table 10:** Comparison of prediction errors between the DVIX and other uncertainty indicators based on time dimension

Asset	EMU (%)	VIX (%)	VOL (%)	USEPU (%)	UKEPU (%)	CNEPU (%)	PCA (%)	PLS (%)	s-FPCA (%)	s-PCA (%)	$T_{\mathrm {OS}}$ (%)
AORD	50.06	51.42	**49.50**	50.55	50.06	50.73	**48.73**	50.48	**45.95**	**47.97**	4061
BFX	53.50	50.83	52.97	53.96	53.23	53.50	52.99	53.55	**49.68**	50.17	4114
BSESN	51.32	50.31	51.25	51.35	51.88	50.89	51.17	50.99	**48.14**	50.15	3926
BVSP	50.50	**49.61**	51.46	51.69	51.89	51.49	50.44	50.77	**49.02**	**48.64**	3939
DJI	53.90	**48.78**	53.66	53.40	53.86	53.45	52.83	53.90	52.47	51.10	4176
FCHI	53.91	50.75	53.23	54.10	54.49	53.81	52.87	54.01	**49.49**	**49.56**	4118
FTMIB	54.66	52.31	53.61	53.81	54.06	54.26	53.96	55.12	**48.95**	**48.85**	1994
FTSE	52.61	**49.94**	52.00	52.22	52.17	52.15	51.48	51.63	**48.93**	**48.98**	4079
GDAXI	53.20	51.90	52.58	53.73	53.86	53.37	52.73	53.49	**48.69**	**48.84**	4083
GSPTSE	53.03	51.01	52.79	52.30	53.00	52.03	51.74	52.60	50.44	51.39	3711
HSI	52.25	**49.92**	51.53	52.81	52.23	52.76	51.25	51.43	**48.87**	**49.41**	3910
IBEX	54.88	51.33	53.63	54.12	53.78	54.42	53.46	54.64	**49.35**	**49.96**	4087
KS11	52.25	51.79	52.15	52.28	52.66	52.28	52.43	50.87	**48.36**	**49.05**	3912
KSE	**49.19**	**49.22**	50.99	**49.45**	**49.71**	**48.93**	**48.02**	**49.69**	50.81	50.57	3840
MXX	52.05	**49.91**	51.35	52.17	52.05	52.32	50.98	51.30	50.11	**49.62**	4031
N225	54.36	52.94	54.39	54.78	54.62	54.86	52.94	52.70	**47.04**	50.31	3850
OMXC20	51.83	50.73	50.23	52.32	50.87	51.54	50.87	52.07	**47.71**	**48.60**	2821
OMXHPI	51.59	51.35	52.22	53.77	53.20	53.59	51.21	52.22	**46.97**	**47.39**	2855
OMXSPI	53.78	52.45	53.29	54.84	54.56	53.75	53.05	53.99	**48.28**	**49.51**	2854
OSEAX	51.24	**49.39**	50.90	52.11	51.71	51.90	50.59	50.59	**47.58**	**47.89**	3823
SSEC	52.34	51.71	51.50	53.20	52.60	51.86	51.97	52.26	**49.19**	**49.61**	3810
SSMI	52.89	50.93	53.36	53.88	53.73	53.63	52.94	52.25	**48.77**	**49.19**	4031
STI	51.97	**49.81**	51.64	51.45	51.36	51.88	50.47	51.22	**48.22**	**47.75**	2132
STOXX50E	52.84	50.85	52.62	52.82	53.18	53.06	52.19	53.06	**48.57**	**48.79**	4116

This table reports the superior probability defined as 18 for comparing the prediction accuracy between the DVIX and other uncertainty measures in the time dimension. If the value is more than 50%, the DVIX has lower prediction errors relative to other uncertainty indexes during more than half of out-of-sample periods. The bold font highlights the values being less than 50%. $T_{\mathrm {OS}}$ donates the out-of-sample size

We noted that the predicted value of DVIX was more often closer to the real value than the other UIs were, although the superiority did not appear to be very significant since the superior probabilities approached 50%. Thus, we further analyzed the (absolute) prediction error sequence to investigate the impacts of the extreme values (from the stability dimension). Table 11 presents the 99%, 95%, and 90% quantiles of the prediction error sequences of UIs after subtracting that of DVIX. The positive (negative) ones denote that the prediction error of DVIX at the quantile was smaller (larger) than those of UIs. We highlighted the negative ones in bold font. The results demonstrated that most UIs exhibited higher extreme prediction errors than DVIX, indicating that DVIX delivered better prediction results since its prediction errors were more stable (exhibiting less-extreme values). Finally, compared with DVIX, we observed that the s-PCA-based index exhibited an advantage and a disadvantage in the time and stability dimensions. This could account for why they exhibited their prediction advantages in different markets.

**Table 11:** Comparison of prediction errors between the DVIX and other uncertainty indicators based on stability dimension

Asset	Quantile (%)	EMU	VIX	VOL	USEPU	UKEPU	CNEPU	PCA	PLS	s-FPCA	**s-PCA**
AORD	99	0.064	0.041	0.051	0.085	0.100	0.093	0.047	0.009	− **0.018**	− **0.028**
	95	0.019	0.007	0.006	0.019	0.020	0.022	0.004	**0.000**	− **0.009**	− **0.013**
	90	0.006	0.003	0.005	0.004	0.004	0.006	0.003	0.008	**0.000**	**0.000**
BFX	99	0.045	0.001	0.045	0.044	0.042	0.045	0.036	0.027	0.030	0.025
	95	0.018	− **0.002**	0.017	0.016	0.018	0.016	0.010	0.016	0.002	− **0.001**
	90	0.009	0.007	0.010	0.013	0.012	0.013	0.009	0.009	0.001	0.001
BSESN	99	− **0.002**	− **0.016**	0.010	0.005	0.000	0.001	− **0.013**	− **0.011**	0.002	0.001
	95	0.010	0.001	0.007	0.008	0.011	0.008	0.006	0.002	**0.000**	0.001
	90	0.005	0.004	0.007	0.006	0.008	0.004	0.005	0.006	0.000	0.000
BVSP	99	0.015	0.024	0.014	0.019	0.021	0.011	0.005	0.018	0.007	0.005
	95	0.005	0.001	0.002	0.005	0.007	0.006	0.002	0.004	− **0.001**	− **0.001**
	90	0.007	0.008	0.008	0.006	0.008	0.007	0.004	0.009	0.002	0.004
DJI	99	0.007	− **0.005**	0.023	0.028	0.030	0.023	0.008	0.010	0.000	**0.000**
	95	0.013	0.006	0.013	0.019	0.016	0.014	0.007	0.010	0.004	0.005
	90	0.009	− **0.010**	0.012	0.011	0.012	0.011	0.007	0.005	0.001	0.003
FCHI	99	0.032	− **0.004**	0.012	0.031	0.025	0.028	0.011	0.012	0.005	0.010
	95	0.021	0.016	0.023	0.025	0.021	0.022	0.018	0.018	0.005	0.008
	90	0.021	0.008	0.017	0.022	0.021	0.020	0.015	0.014	0.002	0.002
FTMIB	99	0.025	− **0.010**	0.016	0.024	0.022	0.020	0.008	0.020	**0.000**	− **0.001**
	95	0.018	0.007	0.020	0.022	0.020	0.025	0.009	0.016	0.003	0.006
	90	0.010	0.010	0.006	0.008	0.008	0.007	0.007	0.005	− **0.001**	− **0.001**
FTSE	99	0.052	0.029	0.047	0.061	0.052	0.057	0.037	0.024	0.023	0.023
	95	0.015	0.013	0.005	0.013	0.014	0.011	0.012	0.014	− **0.008**	− **0.003**
	90	0.008	0.007	0.007	0.011	0.010	0.009	0.005	0.010	− **0.001**	− **0.001**
GDAXI	99	0.022	− **0.017**	0.035	0.028	0.026	0.036	0.008	0.007	− **0.006**	− **0.001**
	95	0.020	0.010	0.016	0.024	0.017	0.018	0.017	0.018	0.001	0.004
	90	0.016	0.012	0.014	0.019	0.020	0.019	0.009	0.014	0.004	0.004
GSPTSE	99	0.005	0.002	0.003	0.020	0.020	0.008	0.003	− **0.011**	− **0.001**	0.001
	95	0.003	− **0.007**	0.002	− **0.002**	− **0.002**	0.000	− **0.009**	− **0.006**	− **0.001**	0.001
	90	0.012	0.012	0.012	0.013	0.013	0.011	0.008	0.011	0.001	0.003
HSI	99	0.010	− **0.002**	0.015	0.020	0.025	0.027	0.004	− **0.007**	**0.000**	− **0.005**
	95	0.011	0.006	0.008	0.009	0.010	0.007	− **0.001**	0.004	**0.000**	**0.000**
	90	0.001	0.006	0.005	0.002	0.003	0.004	0.003	0.005	0.002	− **0.001**
IBEX	99	0.022	− **0.001**	0.019	0.023	0.015	0.018	0.016	0.014	0.007	0.006
	95	0.009	0.004	0.009	0.006	0.009	0.008	0.005	0.004	0.001	0.001
	90	0.004	0.006	0.007	0.009	0.010	0.008	0.001	0.006	− **0.001**	− **0.001**
KS11	99	0.034	− **0.003**	0.034	0.018	0.026	0.025	0.028	0.034	0.017	0.010
	95	0.014	0.012	0.014	0.019	0.017	0.015	0.008	0.016	0.005	0.003
	90	0.009	0.002	0.011	0.011	0.010	0.011	0.007	0.007	0.003	0.000
KSE	99	− **0.010**	− **0.033**	0.005	0.001	0.005	0.001	0.002	− **0.009**	− **0.002**	**0.000**
	95	0.003	0.002	0.002	0.004	0.002	0.003	0.005	0.005	0.001	0.003
	90	− **0.002**	− **0.004**	− **0.001**	0.001	0.000	− **0.003**	− **0.004**	− **0.002**	− **0.001**	**0.000**
MXX	99	0.012	− **0.006**	0.012	0.019	0.021	0.023	− **0.005**	− **0.002**	− **0.005**	− **0.004**
	95	− **0.001**	0.004	0.004	0.001	0.005	− **0.001**	− **0.005**	− **0.002**	0.000	0.003
	90	0.004	0.004	− **0.001**	**0.000**	− **0.002**	0.000	0.004	0.004	− **0.002**	0.000
N225	99	0.029	0.022	0.045	0.050	0.051	0.050	0.030	0.007	− **0.012**	− **0.022**
	95	0.022	0.020	0.017	0.029	0.027	0.023	0.013	0.013	0.000	0.001
	90	0.010	0.007	0.007	0.011	0.012	0.011	0.005	0.006	− **0.001**	− **0.003**
OMXC20	99	− **0.010**	− **0.009**	− **0.020**	− **0.002**	− **0.009**	− **0.003**	− **0.004**	− **0.026**	− **0.008**	0.005
	95	0.015	0.012	0.011	0.016	0.014	0.016	0.009	0.005	− **0.003**	− **0.006**
	90	0.012	0.008	0.013	0.014	0.015	0.015	0.004	0.004	− **0.002**	− **0.002**
OMXHPI	99	− **0.020**	− **0.007**	− **0.008**	0.007	0.009	0.007	− **0.014**	− **0.023**	− **0.006**	− **0.011**
	95	0.014	− **0.016**	0.014	0.007	0.008	0.011	0.002	0.006	− **0.003**	− **0.005**
	90	0.016	0.006	0.023	0.020	0.019	0.016	0.006	0.012	**0.000**	**0.000**
OMXSPI	99	0.027	0.006	0.031	0.023	0.023	0.025	0.013	0.008	0.002	0.004
	95	0.024	0.007	0.025	0.021	0.023	0.022	0.003	0.012	0.000	0.000
	90	0.021	0.009	0.022	0.026	0.023	0.025	0.008	0.014	**0.000**	**0.000**
OSEAX	99	0.043	0.044	0.039	0.046	0.036	0.041	0.010	0.019	− **0.003**	− **0.003**
	95	0.016	0.002	0.027	0.020	0.022	0.023	0.009	0.010	− **0.001**	− **0.002**
	90	0.016	0.004	0.010	0.009	0.011	0.012	0.009	0.012	0.001	0.001
SSEC	99	0.037	0.017	0.039	0.037	0.039	0.044	0.025	0.025	0.000	0.006
	95	0.003	0.008	0.007	0.004	0.003	0.004	0.002	0.003	0.002	0.001
	90	0.009	0.009	0.010	0.008	0.009	0.010	0.009	0.010	0.002	0.002
SSMI	99	0.045	0.009	0.038	0.046	0.046	0.037	0.030	0.026	0.011	0.021 ra>
	95	0.019	0.023	0.018	0.023	0.022	0.023	0.010	0.017	0.000	0.002
	90	0.014	0.010	0.013	0.013	0.014	0.014	0.008	0.014	0.002	0.003
STI	99	0.012	0.006	0.021	0.012	0.011	0.013	0.010	0.007	0.003	**0.000**
	95	− **0.005**	− **0.012**	− **0.003**	− **0.001**	− **0.001**	− **0.001**	0.002	− **0.004**	− **0.005**	− **0.008**
	90	− **0.001**	− **0.003**	0.001	0.002	0.000	0.003	0.003	− **0.002**	0.001	− **0.001**
STOXX50E	99	0.079	− **0.022**	0.060	0.077	0.078	0.077	0.070	0.042	0.035	0.014
	95	0.005	− **0.005**	0.003	0.005	0.008	0.007	0.001	− **0.002**	− **0.002**	0.004
	90	0.018	0.013	0.014	0.017	0.015	0.018	0.013	0.014	0.002	**0.000**

This table reports 99%, 95% and 90% quantiles of the prediction error of uncertainty indexes after minus that of the DVIX, which is to compare the prediction accuracy between the DVIX and other uncertainty measures in the stability dimension. The bold font donates that it has a smaller prediction error at corresponding quantile with respect to that of the DVIX, indicating that the prediction error sequence is more stable

### Comparison of the composite UIs

The empirical results demonstrated that the PCA-based and PLS-based composite UIs demonstrated lower prediction accuracies compared with the s-PCA-based ones. This subsection further discusses the loadings of these dimension-reduction methods to explain the result. Put differently, we analyzed the main contributors of these composite indexes. Dissimilar to the findings of He et al. (2021) and Neely et al. (2014) who employed static analysis to discuss the loadings, we employed dynamic analysis to demonstrate the change in the loadings with time, and this enabled us to observe the changes in the weight over time and prevented particularity. Based on the one-step-ahead rolling (*W*=1000), we calculated the loadings recurrently. Thus, the length of a series of loadings correlated with the out-of-sample size.

#### Time-varying loadings of the PCA factors

**Fig. 3:**
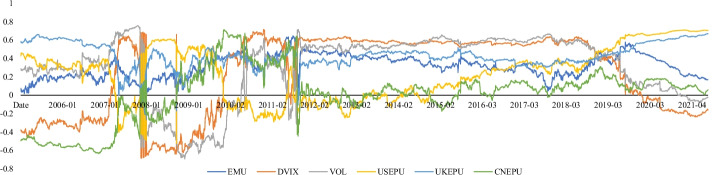
Time-varying loadings of the PCA factors ($W=1000$)

Figure 3 displays the loadings of the PCA factors over time. First, we observed that each loading changed over time, indicating that the contribution of each predictor to the PCA factor was time-varying. Thus, the time-varying analysis was more suitable compared with the static analysis. Moreover, we observed that every single UI exhibited approximate loadings, indicating that each predictor in the PCA component played an equally essential role all the time or sometimes. Notably, EPUs exhibited a limited explanatory power on RVs, which should destroy the predictability of PCA.

#### Time-varying loadings of the PLS factors

**Fig. 4:**
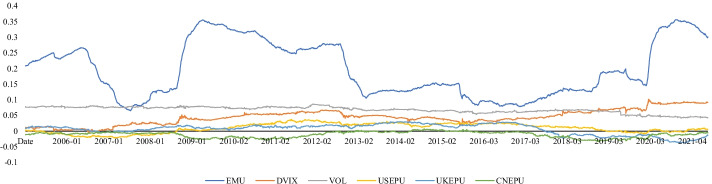
Time-varying loadings of the PLS factors ($W=1000$)

Figure 4 shows that the loadings of the PLS factors were more stable over time compared with those of the PCA method except for EMU. The figure shows that EMU exhibited the largest weight, followed by VOL, DVIX, and the other predictors, indicating that EMU was the main contributor to UI of PLS even though it exhibited time-varying weights. Revisiting the in- and out-of-sample results (Tables 3 and 4), EMU, VOL, and DVIX exerted a significant predictive power on stock volatility in most markets. Thus, PLS performed better than PCA since it could identify and extract the significant predictors and reduce the impacts of the insignificant predictors (EPUs).

#### Time-varying loadings of the s-PCA factors

**Fig. 5:**
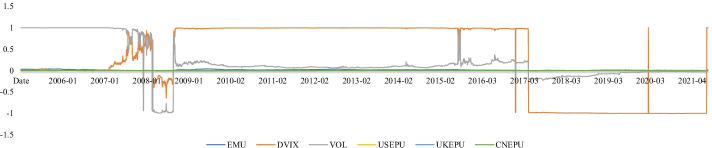
Time-varying loadings of the Scaled-PCA factors ($W=1000$)

Figure 5 shows the time-varying loadings of the s-PCA factors. Interestingly, the figure shows that the s-PCA-based index was mainly constructed by DVIX and VOL since they exhibited a significantly higher weight than the other predictors. VOL dominated other predictors before 2009, while DVIX became the main contributor afterward. For the other predictors (EPUs and EMU), we observed that their weights approached zero over time, indicating that their contributions to the s-PCA-based UIs were limited. Recall that DVIX delivered more outstanding in- and out-of-sample performances than VOL and the other predictors in volatility forecasting. Although PLS and s-PCA were supervised learning techniques, s-PCA could further differentiate between the relative importance of the strong predictors. Put differently, s-PCA could identify the better (worse) predictors, DVIX and VOL, and place more (less) weights on them, while PLS could only identify the powerful predictors but could not arrange reasonable weights. Thus, s-PCA is a more effective dimension-reduction method in the presence of strong and weak predictors.

#### Index performance during the financial crises

**Fig. 6:**
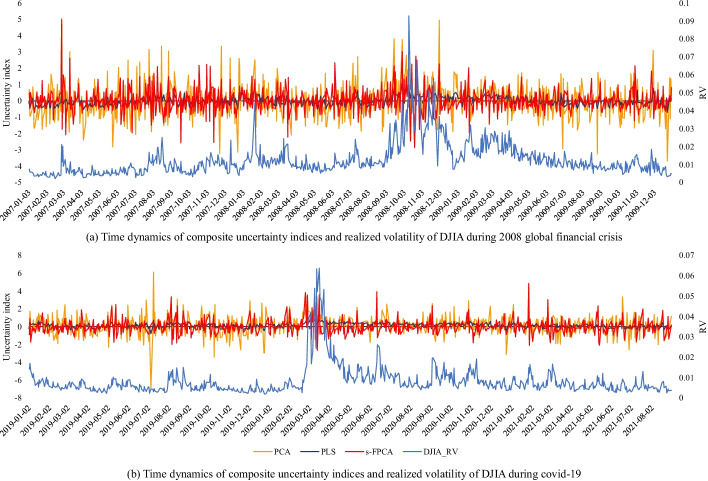
Comparison of uncertainty indices before and after crises

To further observe the differences among composite UIs intuitively, we depicted their time series. Considering that we employed daily data, which were collected within a long period, we demonstrated the time series before and after two well-known crises, namely the 2008 subprime crisis (January 1, 2007, to December 31, 2009) and the 2020 COVID-19 pandemic (January 1, 2020, to the end of the year). For comparison, we added the time dynamics of the U.S. market RV as a reference. Figure 6 shows that the s-PCA-based index (red line) exhibited synchronous and consistent fluctuations with RVs of DJIA (blue line), such as March 3, 2007, November 3, 2008, and August 2, 2019. The PLS-based index (cyan line) exhibited a similar character with the s-PCA-based index only in periods of great fluctuations, such as September 2008 and March 2020. Moreover, it exhibited a small swing, which was not similar to those of RV and the red line with frequent fluctuations, over time. However, the PCA-based index (orange line) fluctuated continually over time, which was just like the random walk process. Although it was challenging to visually capture the relationship between it and RV, we observed that there were no significant differences among PCA-based indexes during financial crises and non-crisis.

In summary, from the loadings and picture analyses, we revealed that the s-PCA method outperformed PCA and PLS owing to two aspects: first, the s-PCA method identified strong predictors and could further place reasonable weight on each predictor. Secondly, compared with the PLS method, s-PCA could solve the over-fitting issue and avoid the incorporation of much noise because it could transform many predictors into orthogonal components Huang et al. (2021), thus reducing the number of variables.

## Conclusion

Uncertainty index is beneficial to decision-making investors and policymakers monitoring market risks. Though enormous efforts have been invested into constructing this index, the method for building one exhibiting a relatively fixed composite and imposing significant impacts on international stock volatilities is still rare, and this study has filled that research gap. We constructed a composite uncertainty index based on the s-PCA method and investigated the high-frequency relationship between the proposed index and stock volatilities in global markets. The proposed index comprehensively captured the uncertainties from the equity-market, investor, and economic-policy levels. More crucially, it was very practical and user-friendly, in reality, for its property of a relatively fixed composite.

The empirical analyses of 23 international stock market volatilities revealed that the proposed index exhibited excellent performances in the in- and out-of-sample predictabilities, and these performances were better and more robust than those of competing models, including the widely employed PCA and PLS methods. This superiority is rational. One reason is that the proposed method reserved the advantage of the PCA method, which avoids adding much noise to the prediction task and reduces the risk of overfitting. The other reason is that the proposed index could not only identify relevant predictors, it also achieved the best use of them by placing more weight on more informative predictors, while the PLS method could not.

Our results exhibit the following practical implications: (i) We availed fixed and valuable indicators for investors and policymakers with keen interests in the international stock markets. These indicators can effectively reflect market risk dynamics. (ii) We established the insignificant high-frequency relationship between EPU and stock volatility, which brings a warning to short-term investors when allocating their wealth. (iii) We discussed the differences among popular dimension-reduction methods that deal with both strong and weak factors, which give a good reference to scholars and practitioners when employing econometric models to investigate market movements.

## Data Availability

The data were derived from public domain resources. The data that support the findings of this study are available on public websites. All the data are available from the authors upon reasonable request.

## Footnotes

1 Realized variance is the square of realized volatility and thus they have the same economic meanings. Although we focus on realized variance in this study, we use the terms stock volatility and realized variance interchangeably.

2 See https://realized.oxford-man.ox.ac.uk/.

3 We split economic expansions and recessions following the NBER, see https://www.nber.org/research/data/us-business-cycle-expansions-and-contractions.

4 Note that the $R_{\mathrm {OS}}^{2}$ is not very large in some cases but statistically significant. This is common since we use high-frequency data in this study. A similar result described in He et al. (2021) reports that the statistically significant $R_{OS}^2$s are 18.38%, 14.53%, and 0.55% for monthly, weekly, and daily frequency, respectively.

5 The result based on the TR statistic is consistent with the TMAX statistic results. The results are not reported owing to space limitations but are available from the authors. For details of MCS test, one can refer to Hansen et al. (2011) and Zhao et al. (2021).
